# EEG-Based Emotion Recognition Using Quadratic Time-Frequency Distribution

**DOI:** 10.3390/s18082739

**Published:** 2018-08-20

**Authors:** Rami Alazrai, Rasha Homoud, Hisham Alwanni, Mohammad I. Daoud

**Affiliations:** 1 School of Electrical Engineering and Information Technology, German Jordanian University, Amman 11180, Jordan; rasha.homoud@gju.edu.jo (R.H.); mohammad.aldaoud@gju.edu.jo (M.I.D.); 2 Faculty of Engineering, University of Freiburg, Freiburg 79098, Germany; hisham.alwanni@pluto.uni-freiburg.de

**Keywords:** emotion recognition, quadratic time-frequency distributions, electroencephalography, time-frequency features, support vector machines, 2D arousal-valence plane

## Abstract

Accurate recognition and understating of human emotions is an essential skill that can improve the collaboration between humans and machines. In this vein, electroencephalogram (EEG)-based emotion recognition is considered an active research field with challenging issues regarding the analyses of the nonstationary EEG signals and the extraction of salient features that can be used to achieve accurate emotion recognition. In this paper, an EEG-based emotion recognition approach with a novel time-frequency feature extraction technique is presented. In particular, a quadratic time-frequency distribution (QTFD) is employed to construct a high resolution time-frequency representation of the EEG signals and capture the spectral variations of the EEG signals over time. To reduce the dimensionality of the constructed QTFD-based representation, a set of 13 time- and frequency-domain features is extended to the joint time-frequency-domain and employed to quantify the QTFD-based time-frequency representation of the EEG signals. Moreover, to describe different emotion classes, we have utilized the 2D arousal-valence plane to develop four emotion labeling schemes of the EEG signals, such that each emotion labeling scheme defines a set of emotion classes. The extracted time-frequency features are used to construct a set of subject-specific support vector machine classifiers to classify the EEG signals of each subject into the different emotion classes that are defined using each of the four emotion labeling schemes. The performance of the proposed approach is evaluated using a publicly available EEG dataset, namely the DEAPdataset. Moreover, we design three performance evaluation analyses, namely the channel-based analysis, feature-based analysis and neutral class exclusion analysis, to quantify the effects of utilizing different groups of EEG channels that cover various regions in the brain, reducing the dimensionality of the extracted time-frequency features and excluding the EEG signals that correspond to the neutral class, on the capability of the proposed approach to discriminate between different emotion classes. The results reported in the current study demonstrate the efficacy of the proposed QTFD-based approach in recognizing different emotion classes. In particular, the average classification accuracies obtained in differentiating between the various emotion classes defined using each of the four emotion labeling schemes are within the range of 73.8%–86.2%. Moreover, the emotion classification accuracies achieved by our proposed approach are higher than the results reported in several existing state-of-the-art EEG-based emotion recognition studies.

## 1. Introduction

Emotions comprise complex mental activities that can influence the physical and psychological behavior of humans during social interactions and decision-making processes. In fact, identifying human emotional states is considered a vital capability towards achieving intelligent and effective social communications in several domains. For example, in the medical domain, discerning the patient’s emotional state can provide caregivers with an indicator about the patient’s mental and physical status [1,2] and the progress of the recovery process [3]. In the education domain, characterizing the students’ emotional state in terms of the level of interest and active participation in the learning process is crucial to achieve effective knowledge transfer and development systems [4]. In the road- and aviation-safety domain, identifying the level of the boredom and vigilance emotional states of the driver/pilot can contribute to reducing the rate of accidents [5].

The literature reveals that researchers have developed various approaches for emotion recognition based on analyzing facial expressions [6,7,8,9], voice [10,11], combined visual and textual modalities [12] and signals related to the autonomic nervous system (ANS), such as the heart rate, skin temperature, respiration patterns, blood volume pressure and galvanic skin response (GSR) [13,14]. Despite the promising results attained for the emotion recognition approaches that are based on facial expressions and voice, these approaches are considered ineffective for subjects suffering from facial paralysis or subjects who have the ability to conceal their real emotional states [3,15,16,17]. Moreover, the requirement of having a camera or a voice recorder all the time to capture the facial expressions and record the voice of the subject limits the applicability of these approaches when the subject is not directly looking at the camera or when the surrounding environment is noisy [2]. In fact, these limitations, which are associated with the facial expression- and voice-based emotion recognition approaches, can be overcome by employing ANS-based emotion recognition approaches. Nonetheless, emotion recognition approaches that are based on analyzing the signals related to the ANS are highly affected by human physical activities. In particular, various human physical activities can produce ANS-related signals that are quite similar to some ANS-related signals generated during several emotional states [2]. For example, performing physical activities can increase the heart rate, the respiratory rate and skin perspiration, which can affect the accuracy of emotion recognition approaches that are based on analyzing the heart rate, respiratory rate and GSR signals.

Recently, researchers have started to utilize physiological signals that are acquired from the central nervous system (CNS) to recognize emotional states. The use of CNS-related physiological signals to recognize human emotions is considered as a remedy to the limitations associated with the facial expression-, voice- and ANS-based emotion recognition approaches. This can be attributed to the fact that the CNS-related physiological signals are not affected by the previously mentioned limitations associated with the other emotion recognition approaches [2]. In this vein, the electroencephalograph (EEG) signals, which provide a quantitative measure of the electrical potentials generated at different locations of the brain in response to a specific stimulus [18], are by far the most commonly-used CNS-related physiological signal for designing emotion recognition systems [2,17]. The widespread use of the EEG signals is attributed to several advantages of the EEG signals, including the non-invasive nature, the high temporal resolution, the high portability of the EEG signal acquisition systems and the low cost of the EEG signal recording systems [19]. Nonetheless, the task of analyzing EEG signals for emotion recognition is considered challenging. This is due to the nonstationary nature of the EEG signals, in which the spectral characteristics of the signals are changing over time [20]. Therefore, analyzing the nonstationary EEG signals in either the time-domain or the frequency-domain is deemed unsuitable [21,22]. In addition, the emotional responses with respect to the same stimulus can vary significantly between subjects, which can introduce large inter- and intra-personal variations in the recorded EEG signals. This in turn can increase the difficulty of discriminating between EEG signals corresponding to different emotional states.

In this study, we propose an EEG-based approach for recognizing human emotions using a set of time-frequency features extracted from a quadratic time-frequency distribution (QTFD). In particular, a QTFD is employed to construct a high resolution time-frequency representation of the EEG signals that can capture the spectral variations of the EEG signals over time. In order to reduce the dimensionality of the constructed QTFD-based time-frequency representation, a set of 13 time- and frequency-domain-based features are extended to the joint time-frequency-domain and extracted from the obtained QTFD-based time-frequency representation. Moreover, the two-dimensional (2D) arousal-valence plane [23] is employed to describe the different emotion classes by developing four emotion labeling schemes of the EEG signals, such that each labeling scheme defines a set of emotion classes. These four emotion labeling schemes are: the one-dimensional two-class labeling scheme (1D-2CLS), one-dimensional three-class labeling scheme (1D-3CLS), two-dimensional four-class labeling scheme (2D-4CLS) and two-dimensional five-class labeling scheme (2D-5CLS). The extracted time-frequency features are used to construct a set of subject-specific support vector machine (SVM) classifiers to classify the EEG signals of each subject into different emotion classes that are defined using each of the four labeling schemes.

In order to validate the performance of the proposed approach, we have utilized a publicly available EEG dataset, namely the dataset for emotion analysis using electroencephalogram, physiological and video signals (DEAP) [24], in which different emotion classes are described in terms of the 2D arousal-valence plane [23]. In fact, the DEAP dataset has been utilized as a benchmark dataset to evaluate the performance of the vast majority of the existing EEG-based emotion recognition approaches, as depicted in Section 4.4. Three performance evaluation analyses were conducted to evaluate the performance of the proposed QTFD-based approach in discriminating between the various emotion classes defined for each of the four labeling schemes. The three performance evaluation analyses are: the channel-based analysis, feature-based analysis and neutral class exclusion analysis. These performance evaluation analyses are designed to quantify the effect of utilizing different groups of EEG channels that cover various regions in the brain, reducing the dimensionality of the extracted time-frequency features, and excluding the EEG signals that correspond to the neutral class, which represents the no-emotion state, to improve the capability of the proposed approach to discriminate between different emotion classes. The reported experimental results demonstrate the efficacy of the proposed approach in discerning different emotion classes based on analyzing EEG signals using QTFD. Moreover, the results presented in this study show that the performance of the proposed approach outperforms those reported in several existing state-of-the-art approaches.

In this study, we aim to contribute to the ongoing research in the field of EEG-based emotion recognition by introducing an EEG-based system that employs a set of time-frequency features extracted from a QTFD-based representation to discriminate between different emotion classes that are defined using various emotion labeling schemes. In fact, we hypothesize that analyzing the EEG signals using QTFD can improve the accuracy of classifying different emotion classes compared with other previously used time-frequency analyses, such as the short time Fourier transform (STFT) and the wavelet transform (WT). The contributions of this study can be summarized in four aspects: (1) to the best of our knowledge, this is the first study that employs the QTFDs to analyze EEG signals for recognizing human emotions; (2) our study proposes a novel set of time-frequency features extracted from the constructed QTFD-based representation to model the different emotion classes encapsulated within the EEG signals; (3) in-depth analysis is carried out to quantify the effect of excluding the EEG signals that correspond to the neutral class on the accuracy of emotion classification; (4) a new emotion labeling scheme is developed to evaluate the performance of the proposed QTFD-based emotion recognition approach, namely the 2D-5CLS. In fact, the 2D-5CLS utilizes the 2D arousal-valence plane to define five different emotion classes, which makes the task of emotion classification more challenging compared with the other labeling schemes employed in previous studies that utilize the 2D arousal-valence plane to define a lower number of emotion classes. To the best of our knowledge, this is the first study that investigates the possibility of discriminating between five emotion classes, which are defined based on the 2D arousal-valence emotion description model.

The remainder of this paper is structured as follows: In Section 2, we provide a detailed description of the utilized EEG dataset, constructed QTFD-based time-frequency representation, extracted time-frequency features, emotion classification model and performance evaluation analyses. In Section 3 and Section 4, we present and discuss the results obtained for each of the three performance evaluation analyses, and we compare the performance of our proposed approach with the performance results reported for several existing state-of-the-art EEG-based emotion recognition approaches. Finally, the conclusion is provided in Section 5.

## 2. Materials and Methods

### 2.1. The DEAP Dataset

In this study, we utilize a publicly available dataset for emotion analysis using EEG signals, namely the dataset for emotion analysis using electroencephalogram, physiological and video signals (DEAP) [24]. DEAP is a multi-modal dataset that comprises EEG and peripheral physiological signals recorded for 32 subjects, including 16 females and 16 males, while watching a set of 40 one-minute video stimuli. The content of each video stimulus was selected to evoke a specific emotional state. For each subject, the recorded EEG signals associated with each video stimulus represent a single trial. The total number of trials within the DEAP dataset is 1280 trials (40 trials per subject), and the length of each trial is 63 s. In particular, each trial starts with a three-second baseline period followed by 60 s of EEG signal recording that corresponds to the subject’s response with respect to a specific video stimulus. After each trial, each subject was asked to quantify his/her emotional response with respect to the displayed video in terms of five emotion description scales, namely valence, arousal, dominance, like/dislike and familiarity scales. The values of the valence, arousal, dominance and like/dislike scales are in the range [1–9], while the values of the familiarity scale are in the range [1–5].

The literature reveals that the vast majority of the existing work related to EEG-based emotion analysis has utilized the arousal and valence scales to describe different emotional states [4,23,24]. Therefore, in this study, we focus on the values of the valence and arousal scales to quantify and describe the various emotional states. In fact, the valence scale quantifies the amount of pleasantness that a subject can feel towards a specific stimulus [24]. Specifically, a valence value that is equal to one indicates a sad or unhappy feeling, while a valence value that is equal to nine indicates a happy or joyful feeling. On the other hand, the arousal scale quantifies the intensity of an elicited emotion that a subject can feel with respect to a specific stimulus [24]. In particular, an arousal value that is equal to one indicates a bored or calm feeling, while an arousal value that is equal to nine indicates excitement or stimulation.

### 2.2. Emotion Labeling Schemes

**Figure 1:**
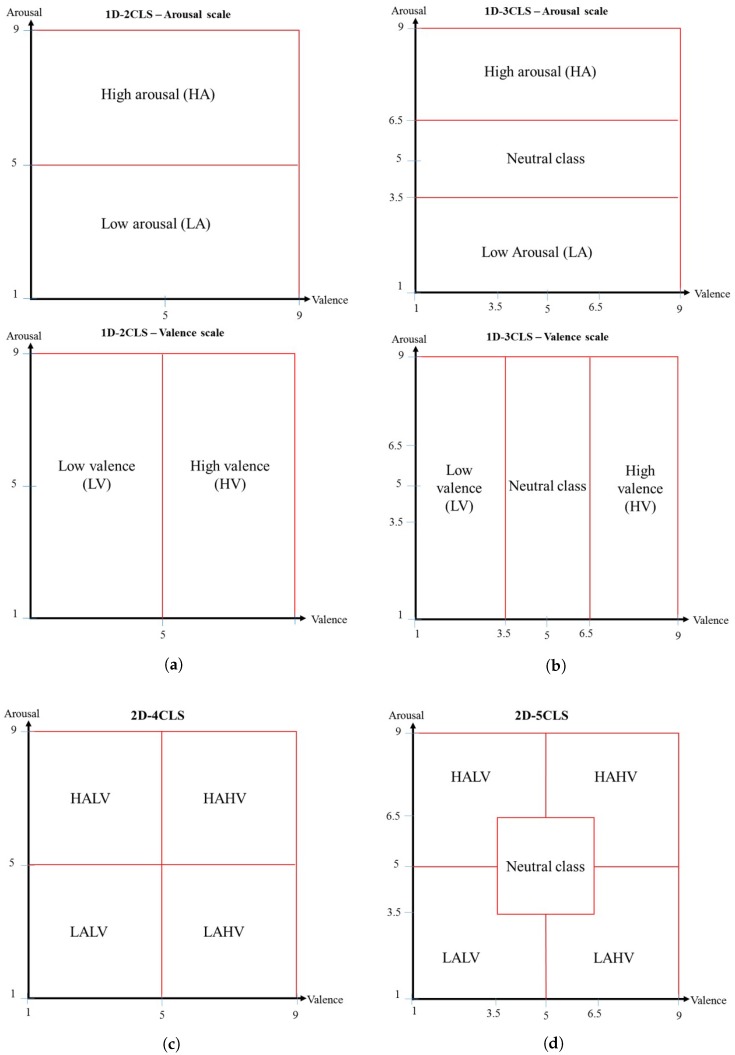
Graphical illustration of the developed four emotion labeling schemes. (**a**) The two emotion classes defined for the arousal scale (**top**) and valence scale (**bottom**) using the 1D-2-class labeling scheme (2CLS). (**b**) The three emotion classes defined for the arousal scale (**top**) and valence scale (**bottom**) using the 1D-3CLS. (**c**) The four emotion classes defined using the 2D-4CLS. (**d**) The five emotion classes defined using the 2D-5CLS.

In this study, various emotion classes are assigned to the EEG signals provided in the DEAP dataset based on the 2D arousal-valence emotion description model [23]. In particular, the emotion class associated with each trail in the DEAP dataset is described using two rating scales, namely the arousal scale and the valence scale, where the values of the arousal and valence scales are in the range [1–9]. Therefore, depending on the number of rating scales utilized to describe an emotion class and the number of intervals defined for each rating scale, different labeling schemes can be developed to label the EEG signals of each trial. In order to investigate the effect of having different labeling configurations of emotion classes on the accuracy of our proposed QTFD-based approach, we have developed four different emotion labeling schemes based on the arousal and valence values provided in the DEAP dataset. The developed emotion labeling schemes can be summarized as follows:

(A) The one-dimensional two-class labeling scheme (1D-2CLS) [17,24,25,26,27,28,29]:
  In this labeling scheme, the arousal and valence scales are used independently to define two emotion classes for each scale. Specifically, for each trial in the DEAP dataset, if the associated arousal value is greater than five, then the trial is assigned to the high arousal (HA) emotion class. Otherwise, the trial is assigned to the low arousal (LA) emotion class. Similarly, for each trial in the DEAP dataset, if the associated valence value is greater than five, then the trial is assigned to the high valence (HV) emotion class. Otherwise, the trial is assigned to the low valence (LV) emotion class. Figure 1a illustrates the emotion classes defined based on the 1D-2CLS.
(B) The one-dimensional three-class labeling scheme (1D-3CLS) [4,28,29,30,31]:
  This emotion labeling scheme utilizes the arousal and valence scales independently to define three emotion classes for each scale. In particular, using the arousal scale, a trial is assigned to the high arousal (HA) emotion class, the neutral emotion class or the low arousal (LA) emotion class depending on whether the associated arousal value is within the interval [6.5–9], (3.5–6.5) or [1–3.5], respectively. Similarly, using the valence scale, a trial is assigned to the high valence (HV) emotion class, the neutral emotion class or the low valence (LV) emotion class depending on whether the associated valence value is within the interval [6.5–9], (3.5–6.5) or [1–3.5], respectively. Figure 1b illustrates the emotion classes defined based on the 2D-3CLS.
(C) The two-dimensional four-class labeling scheme (2D-4CLS) [32,33]:
  This emotion labeling scheme utilizes the 2D arousal-valence plane, which was proposed by Russell [23], to describe and quantify various emotional states. In particular, using the 2D arousal-valence plane, an emotional state can be viewed as a point in the 2D plane defined by the axes of the valence scale and the arousal scale, such that the arousal and valence scales are represented by the vertical and horizontal axes, respectively, of the 2D plane. Therefore, the 2D arousal-valence plane can be divided into four quadrants, where each quadrant represents a specific emotion class. The emotion classes defined based on the 2D-4CLS are: the high arousal/high valence (HAHV), low arousal/high valence (LAHV), low arousal/low valence (LALV) and high arousal/low valence (HALV) emotion classes. The term “low” in each of the four defined emotion classes indicates that the arousal value or the valence value is less than five, while the term “high” indicates that the arousal value or the valence value is greater than five. Figure 1c illustrates the emotion classes defined based on the 2D-4CLS.
(D) The two-dimensional five-class labeling scheme (2D-5CLS):
  In this labeling scheme, we extend the 2D-4CLS to include the neutral emotion class, which represents the no-emotion state. In particular, we divide the 2D arousal-valence plane into five regions, where each region represents a specific emotion class. The emotion classes defined based on the 2D-5CLS are: the HAHV, LAHV, LALV, HALV and neutral emotion classes. The neutral emotion class is employed to represent the trials in which the arousal and valence values fall within the interval (3.5–6.5). Figure 1d illustrates the emotion classes defined based on the 2D-5CLS.

Figure 1 provides a graphical illustration of the defined emotion classes using each of the four emotion labeling schemes. In addition, Table 1 shows the number of trials in the DEAP dataset associated with each emotion class defined based on the 1D-2CLS, 1D-3CLS, 2D-4CLS and 2D-5CLS labeling schemes, respectively.

### 2.3. EEG Signals Acquisition and Preprocessing

The EEG signals were recorded using the BioSemi ActiveTwo system (BioSemi B.V., Amsterdam, The Netherlands) at a sampling frequency of 512 Hz using 32 Ag/AgCl electrodes that were arranged according to the 10–20 international electrode placement system [34]. In this study, we have utilized the preprocessed EEG signals provided by the DEAP dataset. In fact, the procedures that were applied to obtain the preprocessed EEG signals of the DEAP dataset can be summarized as follows. First, the raw EEG signals were downsampled to 128 Hz. Then, the electrooculography (EOG) artifacts, which were generated from eye blinking, were reduced using the blind source separation technique [24]. In addition, the electromyography (EMG) artifacts in the high frequencies, which are generated by muscle activities, were reduced by applying a bandpass frequency filter that had a bandwidth of 4–45 Hz [24]. Finally, the EEG signals were common averaged referenced.

### 2.4. Time-Frequency Analysis of EEG Signals

Time-domain signals can be generally categorized into two main groups: stationary signals and nonstationary signals [35]. Stationary signals have spectral components and statistical properties that are time-invariant. On the other hand, nonstationary signals, such as EEG signals [18,36,37], have spectral characteristics that vary over time [20]. This implies that analyzing nonstationary signals in either the time-domain or frequency-domain is considered inadequate [21,22]. In fact, using a joint time-frequency-domain can capture the time-varying spectral components of nonstationary signals. In this vein, the literature reveals that researchers have employed different time-frequency analysis methods to construct time-frequency representations (TFRs) of nonstationary signals, such as the short-time Fourier transform (STFT) [38] and wavelet transform (WT) [39]. However, STFT-based TFRs have a low resolution in either the time-domain or the frequency-domain, which limits the ability to utilize the STFT method to analyze nonstationary signals [35]. In addition, TFRs that are constructed using the WT method, including the discrete wavelet transform (DWT) and the continuous wavelet transform (CWT), are not invariant to frequency-shift and have a non-uniform resolution throughout the time-frequency plane (TFP) [35].

In this study, we hypothesize that the use of quadratic time-frequency distributions (QTFDs) to construct a TFR of the EEG signals has the potential to provide discriminant features of EEG signals that can enhance the classification accuracy of different emotional states. In fact, QTFDs have been successfully employed to analyze EEG signals in other fields, such as seizure detection [21,40] and decoding motor imagery tasks [18]. A QTFD is a nonlinear transform that maps a time-domain signal into a joint time-frequency-domain that has an excellent resolution in both time and frequency. Moreover, QTFDs are considered time- and frequency-shift-invariant [35,40,41]. Therefore, analyzing the EEG signals using QTFDs can address the limitations associated with other time-frequency analysis methods, such as STFT and WT.

To compute the QTFD of EEG signals, we have utilized a sliding window to divide the EEG signal of each channel into a set of overlapped EEG segments. Therefore, the total number of window positions per each trial is 29 positions. In fact, the number of positions per trail is computed by subtracting one from the ratio between the number of samples per trial after removing the 3-s baseline period, which is 60 s × 128 samples per second, and the overlap size, which is 256 samples. In particular, the number of samples in each EEG segment was set to 512 samples, and the overlap between any two consecutive EEG segments was set to 256 samples. Then, for each EEG segment, z(t), we constructed a TFR by computing the QTFD using the following steps [18,35,36]:

I Compute the analytic signal, a(t), of the real-valued signal, z(t), as follows:
  (1) a(t)=z(t)+jHT{z(t)},
  where HT{·} is the Hilbert transform [42].
II Calculate the Wigner–Ville distribution (WVD) of a(t) as follows:
  (2) $$WVD_{a}\left(t,f\right)=\int_{-\infty }^{\infty }a\left(t+\frac{\tau }{2}\right)a^{*}\left(t-\frac{\tau }{2}\right)e^{-j2\pi \tau f}\partial \tau ,$$
  where $WVD_{a}\left(t,f\right)$ and $a^{*}\left(\cdot \right)$ represent the WVD and the complex conjugate of a(t), respectively.
III Convolve the obtained $WVD_{a}\left(t,f\right)$ with a time-frequency smoothing kernel, χ(t,f), as follows:
  (3) $$\varrho_{a}\left(t,f\right)=\int_{-\infty }^{\infty }\int_{-\infty }^{\infty }WVD_{a}\left(\phi ,\tau \right)\chi \left(\phi ,\tau \right)e^{-j2\pi f\tau -j2\pi t\phi }\partial_{\tau }\partial_{\phi },$$
  where $\varrho_{a}\left(t,f\right)$ represents the QTFD of a(t). In fact, the type of constructed QTFD is determined based on the utilized time-frequency kernel. For example, the time-frequency kernel χ(t,f)=1 is used to construct the WVD, which is considered a QTFD [36]. The WVD produces a TFR that has an excellent resolution in the time- and frequency-domain. Nonetheless, the WVD-based TFR contains cross-terms that may distort the signal components [18,35,41] and, hence, increases the difficulty to interpret the obtained TFR visually. Therefore, in this study, we employ an exponential time-frequency kernel to reduce the effect of the cross-terms and maintain an adequate resolution in both the time- and frequency-domain. The employed exponential kernel is defined as follows:
  (4) $$\chi \left(t,f\right)=\exp\left(-\frac{t^{2}f^{2}}{\beta^{2}}\right),$$
  where β>0 is a parameter that controls the suppression of the cross-terms, and its value is experimentally selected to be 0.5. The QTFD constructed using the kernel defined in Equation (4) is known as the Choi–Williams distribution (CWD) [43].

**Figure 2:**
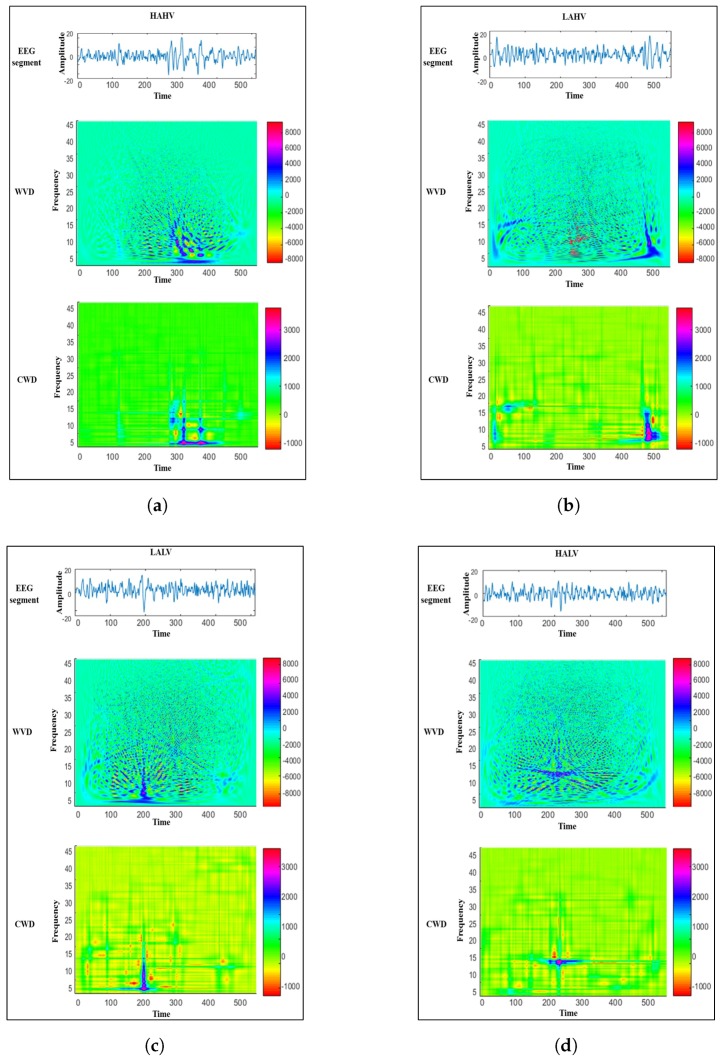
Top view images of the constructed time-frequency representations (TFRs) for four EEG segments that are labeled using the 2D-4CLS. (**a**) The Wigner–Ville distribution (WVD)- and Choi–Williams distribution (CWD)-based TFRs computed for an EEG segment that belongs to the HAHV emotion class. (**b**) The WVD- and CWD-based TFRs computed for an EEG segment that belongs to the LAHV emotion class. (**c**) The WVD- and CWD-based TFRs computed for an EEG segment that belongs to the LALV emotion class. (**d**) The WVD- and CWD-based TFRs computed for an EEG segment that belongs to the HALV emotion class. The time axis represents the indices of the samples within the EEG segment, while the frequency axis represents the frequency components within the EEG segment. The color map located to the right of each plot represents the values of the computed WVD and CWD at each point in the time-frequency plane.

Figure 2 shows the top view of the TFRs computed for four EEG segments that are labeled using the 2D-4CLS. The TFRs in the figure aim to illustrate the variations of the frequency content of each EEG segment as a function of time. Moreover, the provided TFRs illustrate how the CWD can reduce the cross-terms in comparison with the WVD, which in turn generates more distinguishable TFPs that can be used for decoding emotional states. Therefore, in this study, the CWD is employed to construct a TFR for each EEG segment. The implementation of the CWD was carried out using the HOSA toolbox [44]. The dimensionality of the computed TFR of each EEG segment, which is obtained based on computing the CWD of the analytic signal associated with the EEG segment, is equal to M=512×N=1024, where *M* and *N* represent the number of time-domain and frequency-domain samples, respectively.

### 2.5. CWD-Based Time-Frequency Features

In order to reduce the dimensionality of the constructed CWD-based TFR, at each window position, we have extracted a set of 13 time-frequency features from the computed CWD of each EEG segment (i.e., the EEG signal encapsulated within the current window position of a specific EEG channel). Then, the extracted time-frequency features from all EEG segments at a specific window position are concatenated to construct a feature vector. Table 1 provides the total number of feature vectors for the 32 subjects and the mean number of feature vectors per each individual subject computed for each emotion class, where the emotion classes are defined using the four emotion labeling schemes described in Section 2.2. The extracted time-frequency features are computed based on extending a set of time- and frequency-domain features into the joint time-frequency-domain. In particular, eight time-domain features are extended into the joint time-frequency-domain and computed based on the constructed CWD-based TFR of each EEG segment [18]. These features include the mean (μ), variance (σ), skewness (γ), kurtosis (κ), sum of the logarithmic amplitudes (SLA), median absolute deviation (MAD), root mean square value (RMS) and inter-quartile range (IQR), where μ, σ, γ and κ are the first-, second-, third- and fourth-order moments, respectively, and represent the higher order statistics (HOS) of the computed QTFD. Table 2 provides a detailed description of the extracted time-frequency features that were obtained by extending the time-domain features. In addition, five frequency-domain features were extended into the joint time-frequency-domain and computed based on the constructed CWD-based TFR of each EEG segment [18]. These features include the flatness (FLS), flux (FLX), spectral roll-off (SRO), normalized Renyi entropy (NRE) and energy concentration (EC). Table 3 provides a detailed description of the five extracted time-frequency features obtained by extending the frequency-domain features. Further details regarding the construction procedure and the physical interpretation of the extracted time-frequency features can be found in our previous work [18].

### 2.6. Emotion Classification

Over the past decade, researchers have employed various classifiers to recognize various emotion classes based on features that are extracted from the EEG signals. Among these classifiers, the support vector machine (SVM) classifier with the Gaussian radial basis function (RBF) kernel has achieved classification and generalization results that outperform other state-of-the-art classifiers, such as the k-nearest neighbors (k-NN) [2,32,45], random forest [4], naive Bayes [28] and neural networks classifiers [40,46,47].

In this study, we employ SVM classifiers with the RBF kernel to determine the emotion classes associated with the feature vectors extracted from the EEG segments. Specifically, depending on the utilized emotion labeling scheme, which are depicted in Section 2.2, the number of emotion classes associated with each emotion labeling scheme varies from 2–5. Therefore, the choice of using a binary or multi-class SVM classifier depends on the utilized emotion labeling scheme. Moreover, to address the large variations in the responses of the subjects with respect to the same video stimulus, we employ a subject-specific classifier rather than constructing one classifier for all the subjects [24]. Consequently, for each subject, we construct a binary or multi-class SVM classifiers depending on the employed emotion labeling scheme. More specifically, using the arousal scale in the 1D-2CLS, each feature vector belongs to the HA or LA emotion class. Similarly, using the valence scale in the 1D-2CLS, each feature vector belongs to the HV or LV emotion class. Therefore, using the use of the 1D-2CLS requires the construction of two binary SVM classifiers for each subject. The first binary SVM classifier classifies the feature vectors into HA and LA classes, while the second binary SVM classifier classifies the feature vectors into into HV and LV classes. For the 1D-3CLS, each feature vector is assigned to one of the three emotion classes defined based on the arousal scale, namely the HA, neutral and LA emotion classes. Similarly, each feature vector is assigned to one of the three emotion classes defined based on the valence scale, namely the HV, neutral and LV emotion classes. Accordingly, the use of the 1D-3CLS implies the need to construct two multi-class SVM classifiers for each subject. The first classifier classifies each feature vector into one of the three arousal-related emotion classes defined based on the 1D-3CLS, including the HA, neutral and LA classes, while the second classifier classifies each feature vector into one of the three valence-related emotion classes defined based on the 1D-3CLS, including the HV, neutral and LV classes. For the 2D-4CLS, each feature vector is assigned to one of the four emotion classes defined based on the 2D-4CLS, namely the HAHV, LAHV, LALV and HALV emotion classes. As a result, for each subject, we construct a multi-class SVM classifier to classify each feature vector into one of the four emotion classes defined on the 2D-4CLS. Finally, for the 2D-5CLS, each feature vector is assigned to one of the five emotion classes defined based on the 2D-5CLS, namely the HAHV, LAHV, LALV, HALV and neutral emotion classes. Consequently, for each subject, we construct a multi-class SVM classifier to classify each feature vector into one of the five emotion classes defined based on the 2D-5CLS.

To construct the multi-class SVM classifiers, which are employed in the 1D-3CLS, 2D-4CLS and 2D-5CLS labeling schemes, we utilize a one-against-one approach in which a binary SVM classifier is trained for each pair of classes, and a voting procedure among all the trained binary classifiers is applied to classify the feature vectors [48,49]. The use of the one-against-one approach to implement the multi-class SVM classifiers can reduce the possibility of generating imbalanced training sets compared to the one-against-all approach [49]. Moreover, in order to achieve the best performance for each of the constructed SVM classifiers, we perform a two-dimensional grid-based search to find the values of the RBF kernel parameter (ω>0) and the SVM regularization parameter (C>0) that minimize the classification error of each SVM classifier. The implementation of the binary and multi-class SVM classifiers is carried out using the LIBSVM [50].

### 2.7. Evaluation Analyses and Metrics

**Figure 3:**
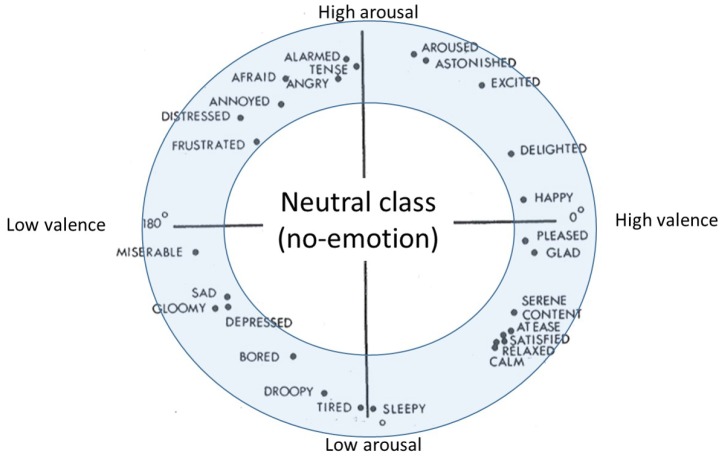
The circumplex model for emotion description that shows the arrangement of the emotional states around the circumference of the 2D arousal-valence plane [23].

In order to evaluate the performance of the proposed QTFD-based features in recognizing different emotion classes, we have developed three performance evaluation analyses:

A Channel-based analysis:
  In this analysis, we investigate the effect of utilizing various groups of EEG channels, which cover different regions of the brain, on the accuracy of recognizing the emotion classes defined based on the four emotion labeling schemes. Recently, several studies have indicated that the frontal, prefrontal, temporal, parietal and occipital regions of the brain are involved in emotional responses [25,32,51,52,53,54,55,56,57,58]. In particular, Mohammadi et al. [58] utilized five pairs of electrodes that cover the frontal and frontal parietal regions of the brain, where these pairs are F3-F4, F7-F8, FC1-FC2, FC5-FC6 and FP1-FP2, to recognize emotional states defined based on the 2D arousal-valence plane. In another study by Zhuang et al. [25], the prefrontal, parietal, occipital and temporal regions were found to have an important role in emotion recognition. These regions of the brain are covered by the following pairs of electrodes: AF3-AF4, P3-P4, P7-P8, CP5-CP6, O1-O2, and T7-T8. Therefore, in this study, we have selected 11 symmetrical pairs of EEG channels out of the 16 pairs of EEG channels provided in the DEAP dataset. The selected pairs of electrodes cover the parietal region (P3-P4, P7-P8 and CP5-CP6), frontal region (F3-F4, F7-F8, FC1-FC2, FC5-FC6, AF3-AF4 and FP1-FP2), temporal region (T7-T8) and occipital region (O1-O2). Table 4 presents the brain regions covered by the selected 11 pairs of EEG channels.
  To study the effect of the utilized different EEG channels on the accuracy of decoding emotion classes, the selected 11 pairs of EEG channels were organized into four different configurations to perform the analysis. Specifically, in the first configuration, denoted by $C_{1}$, we investigate the effect of utilizing each symmetrical pair of EEG channels independently on the accuracy of decoding the emotion classes defined in each emotion labeling scheme. In the second configuration, denoted by $C_{2}$, we study the effect of utilizing 12 EEG channels that are located in the frontal and temporal regions of the brain on the accuracy of recognizing the emotion classes of each emotion labeling scheme. In the third configuration, denoted by $C_{3}$, we explore the effect of utilizing eight EEG channels that are located in the parietal and occipital regions of the brain on the accuracy of recognizing the emotion classes of each emotion labeling scheme. Finally, in the fourth configuration, denoted by $C_{4}$, we study the effect of utilizing all the selected 22 EEG channels on the accuracy of recognizing the emotion classes of each emotion labeling scheme. Table 5 summarizes the aforementioned four configurations and shows the EEG channels comprised within each configuration.
  To implement this evaluation analysis, for each subject, we built an SVM classifier to perform the classification analysis associated with each emotion labeling scheme using the time-frequency features extracted from the EEG channels in each configuration. Specifically, for each symmetrical pair of EEG channels in $C_{1}$, we build a SVM model for each emotion labeling scheme. The dimensionality of the feature vectors extracted from each symmetrical pair of EEG channels in $C_{1}$ is equal to 26 (13features×2channels). Similarly, for each group of EEG channels defined in $C_{2}$, $C_{3}$ and $C_{4}$, we built an SVM model for each emotion labeling scheme. The dimensionality of the feature vectors extracted from the EEG channels in $C_{2}$, $C_{3}$ and $C_{4}$ is 156 (13features×12channels), 104 (13features×8channels) and 286 (13features×22channels), respectively.
B Feature-based analysis:
  In this analysis, we investigate the effect of reducing the dimensionality of the extracted feature vectors on the accuracy of recognizing the emotion classes defined based on the four emotion labeling schemes. In particular, we utilize the minimal redundancy maximum relevance (mRMR) [59] algorithm to reduce the dimensionality of the constructed feature vectors. The mRMR algorithm utilizes the mutual information to select a subset of features that has the maximum correlation with a specific emotion class and the minimum correlation between the selected features [59,60]. The selected subset of features is ranked according to the minimal-redundancy-maximal-relevance criterion. Previous studies [61,62] indicated that using the mRMR algorithm to select features for emotion classification applications can outperform other feature selection algorithms, such as the ReliefFfeature selection algorithm [63]. In this work, we employ the mRMR algorithm to construct four feature selection scenarios, namely the top 5%, 25%, 50% and 75% scenarios. In particular, the mRMR algorithm is used to reduce the size of the extracted feature vectors by selecting the top 5%, 25%, 50% and 75% of the features that satisfy the minimal-redundancy-maximal-relevance criterion [62]. Then, we study the effect of utilizing the features obtained using each of the feature selection scenarios on the accuracy of recognizing the emotion classes of each emotion labeling scheme. For the purpose of this evaluation analysis, we utilize the results obtained from the channel-based evaluation analysis to apply the mRMR feature selection algorithm on the feature vectors extracted from the EEG channels associated with the EEG channel configuration that achieves the best classification performance.
C Neutral class exclusion analysis:
  In this evaluation analysis, we study the effect of excluding the samples that correspond to the neutral class, which are defined in the 1D-3CLS and 2D-5CLS, on the accuracy of decoding the remaining non-neutral emotion classes. According to Russell [23], emotional states are organized in a circular configuration around the circumference of the 2D arousal-valence plane, as depicted in Figure 3. This implies that the region corresponding to the neutral class, which is the area bounded by the interval (3.5–6.5) on the arousal scale and the interval (3.5–6.5) on the valence scale, does not describe emotional states effectively [4]. Therefore, in this evaluation analysis, we exclude the feature vectors extracted from the trials that are falling within the region that represents the neutral class on the 2D arousal-valence plane. To implement this evaluation analysis, we re-perform the previous two evaluation analyses, namely the channel- and feature-based analyses, after excluding the feature vectors that belong to the neutral class.

In order to quantify the performance of our proposed approach for each of the three aforementioned evaluation analyses, we have utilized a ten-fold cross-validation (CV) procedure to train and test the constructed SVM classifiers. In particular, for each subject, 90% of the feature vectors are used for training, while the remaining 10% are used for testing. This procedure is repeated 10 times to ensure that all the feature vectors are used for testing. Then, for each subject, the average classification performance is computed over the ten train-test repetitions (i.e., the ten repetitions of the ten-fold cross-validation procedure) in terms of two standard evaluation metrics, namely the accuracy (acc) and $F_{1}$-measure $\left(F_{1}\right)$ [18,64,65,66]. Finally, we have computed the average values of the accuracies and F1–measures over the 32 subjects of the DEAP dataset and report these average values in Section 3. The utilized evaluation metrics can be computed as follows:

(18) $$acc=\frac{\left(\mathrm{tp}+\mathrm{tn}\right)}{\left(\mathrm{tp}+\mathrm{tn}+\mathrm{fp}+\mathrm{fn}\right)}\times 100\%,$$

(19) $$F_{1}=\frac{2\times \mathrm{tp}}{2\times \mathrm{tp}+\mathrm{fp}+\mathrm{fn}}\times 100\%,$$

where tp, tn, fp and fn represent the numbers of true positive cases, true negative cases, false positive cases and false negative cases, respectively. The $F_{1}$ is an evaluation metric that can be used to assess the performance of the classifiers when the number of samples per different classes is imbalanced. In fact, Table 1 shows that the number of samples for the different classes in the DEAP dataset is imbalanced. Therefore, using the $F_{1}$ provides a more accurate assessment of the performance of the classifiers compared with the standard accuracy rate [24,66].

## 3. Results

In this section, we present the results obtained for each of the three evaluation analyses, which are described in Section 2.7, computed using the four emotion labeling schemes, as presented in Section 2.2.

### 3.1. Results of the Channel-Based Evaluation Analysis

Table 6 presents the average acc and $F_{1}$ values computed for each combination of emotion labeling scheme and EEG channel configuration. In particular, using the 1D-2CLS and $C_{1}$, the highest average acc and $F_{1}$ values achieved in discriminating between the HA and LA classes were 75.9% and 66.7%, respectively, which were obtained using the time-frequency features extracted from the symmetrical pair of EEG channels O1-O2.Moreover, the highest average acc and $F_{1}$ values achieved in discriminating between the HV and LV classes were 73.9% and 69.7%, respectively, which were obtained using the time-frequency features extracted from the symmetrical pair of EEG channels AF3−AF4. Using the 1D-3CLS and $C_{1}$, the highest average acc and $F_{1}$ values achieved in discriminating between the HA, neutral and LA classes were 67.0% and 44.9%, respectively, and the highest average acc and $F_{1}$ values achieved in discriminating between the HV, neutral and LV classes were 65.6% and 51.6%, respectively. These results were obtained using the time-frequency features extracted from the symmetrical pair of EEG channels T7-T8. Using the 2D-4CLS and $C_{1}$, the highest average acc and $F_{1}$ values attained in discriminating between the HVHA, HVLA, LVLA and LVHA emotion classes were 60.5% and 49.4%, respectively. Furthermore, using the 2D-5CLS and $C_{1}$, the highest average acc and $F_{1}$ values attained in discriminating between the HVHA, HVLA, LVLA, LVHA and neutral emotion classes were 57.9% and 45.3%, respectively. The best results reported for the 2D-4CLS and 2D-5CLS were obtained using the time-frequency features extracted from the symmetrical pair of EEG channels T7-T8 in $C_{1}$. In fact, the results reported for each emotion labeling scheme based on $C_{1}$ show that the obtained accuracies using different symmetrical pairs of EEG channels were relatively close to each other. This can be attributed to the fact that different regions located in the left and right hemispheres of the brain were involved during the experience of emotions [4,56].

Table 6 also shows that the average acc and $F_{1}$ values obtained for each emotion labeling scheme using the time-frequency features extracted from the EEG channels of $C_{2}$ were higher than the results obtained using the time-frequency features extracted from the EEG channels of $C_{1}$ and $C_{3}$. In addition, the average acc and $F_{1}$ values obtained for each emotion labeling scheme using the time-frequency features extracted from the EEG channels of $C_{4}$ outperformed the results obtained using the time-frequency features extracted from the other three EEG channel configurations. The results obtained based on $C_{2}$, $C_{3}$ and $C_{4}$ complied with the findings reported in several previous studies [4,54,62], which have shown that the frontal, prefrontal and temporal lobes of the brain play a major role in affective reactions and emotion regulation [4]. This in turn explains the higher accuracies achieved based on $C_{2}$, which covers the frontal and temporal regions of the brain, compared with the accuracies obtained using $C_{3}$, which covers the parietal and occipital regions of the brain. The higher performance achieved using $C_{4}$, which covers the frontal, temporal, parietal and occipital regions of the brain, compared with $C_{1}$, $C_{2}$ and $C_{3}$ can be attributed to the volume conductor effect on the EEG signals [67], which implies that the electrical activities produced within a small region of the brain are spatially propagated to other regions, and consequently, these activities are captured by the sparsely-distributed electrodes on the scalp [18,67,68].

### 3.2. Results of the Feature-Based Evaluation Analysis

The results presented in Section 3.1 indicate that the time-frequency features extracted from the EEG channels of $C_{4}$ achieved the best classification performance for all the emotion labeling schemes. Therefore, in this subsection, we have selected $C_{4}$ to conduct the feature-based analysis. Table 7 presents the results of the feature-based evaluation analysis obtained for each labeling scheme using the time-frequency features extracted from the EEG channels in $C_{4}$.

The results presented in Table 7 show that, for all the emotion labeling schemes, the best average acc and $F_{1}$ values were achieved using the top 25% ranked features. In particular, for the 1D-2CLS, the top 25% of the features achieved average $acc/F_{1}$ values of 86.6%/83.8% and 85.8%/82.4% in discriminating between the HA/LA and HV/LV classes, respectively. For the 1D-3CLS, the top 25% of the features achieved average $acc/F_{1}$ values of 78.8%/65.8% and 77.8%/70.6% in discriminating between the HA/neutral/LA and HV/neutral/LV classes, respectively. Similarly, for the 2D-4CLS and 2D-5CLS, the top 25% of the features achieved average $acc/F_{1}$ values of 75.1%/68.8% and 73.8%/61.9% in discriminating between the emotion classes defined based on the 2D-4CLS and 2D-5CLS, respectively. Table 8 shows the average accuracy and standard deviation values obtained for each subject using the top 25% of the features extracted from the EEG channels in C4. In particular, for each subject, we have computed the average accuracy and standard deviation over the ten train-test repetitions of the employed ten-fold cross-validation procedure. The results presented in Table 8 validate the capability of our-proposed QTFD-based approach in recognizing different emotion classes for each subject in the DEAP dataset.

### 3.3. Results of the Neutral Class Exclusion Analysis

In this section, we present the results obtained for our proposed approach after excluding the feature vectors that correspond to the neutral class in the 1D-3CLS and 2D-5CLS. In particular, for each subject, we utilized the feature vector obtained after eliminating the feature vectors that correspond to the neutral class in the 1D-3CLS to construct two SVM classifiers that discriminate between the HA/LA classes and the HV/LV classes. Similarly, for each subject, we employed the feature vector obtained after excluding the feature vectors that correspond to the neutral class in the 2D-5CLS to construct an SVM classifier that discriminated between the four classes: HAHV, LAHV, LALV and HALV. Table 9 provides the results of applying the channel-based evaluation analysis, described in Section 2.7, for the 1D-3CLS and 2D-5CLS after excluding the feature vectors that correspond to the neutral class.

The results presented in Table 9 show that the average acc and $F_{1}$ values obtained for the 1D-3CLS and 2D-5CLS using the time-frequency features extracted from the EEG channels of $C_{4}$ outperformed the results obtained using the time-frequency features extracted from the other three EEG channel configurations. In fact, these results adhere to the findings presented in Section 3.1, which implies that using the EEG channels of $C_{4}$ provides the best emotion classification performance. Moreover, the acc and $F_{1}$ values reported for the 1D-3CLS and 2D-5CLS using $C_{4}$, which are reported in Table 9, show a significant improvement compared with the acc and $F_{1}$ values presented in Table 6 for the 1D-2CLS, 1D-3CLS, 2D4CLS and 2D-5CLS using $C_{4}$. This increase in the acc and $F_{1}$ values can be attributed to the exclusion of the neutral samples, which reduces the confusion between the different emotion classes comprised in the four labeling schemes.

Table 10 shows the results of the feature-based evaluation analysis, described in Section 2.7, computed for the 1D-3CLS and 2D-5CLS after excluding the feature vectors that correspond to the neutral class. The feature vectors employed in this analysis were extracted from the EEG channels in $C_{4}$, which obtained the best classification performance as indicated in Table 9. The results presented in Table 10 show that the best average acc and $F_{1}$ values computed for the 1D-3CLS and 2D-5CLS labeling schemes were achieved using the top 25% ranked features. These results, which were obtained after excluding the neutral samples, agree with the results presented in Section 3.2, in which the best classification performance was obtained using the top 25% of the time-frequency features extracted from the EEG channels of $C_{4}$.

## 4. Discussion

In this section, we provide a detailed discussion of the results presented in Section 3. Moreover, we compare the performance of our proposed approach with other existing EEG-based emotion recognition approaches that utilize the DEAP dataset.

### 4.1. Channel-Based Evaluation Analysis

The results in Table 6 indicate that the values of the accuracy and $F_{1}$-measure obtained for the 1D-3CLS and 2D-5CLS have been generally decreased compared with the accuracy and $F_{1}$-measure values obtained for the 1D-2CLS and 2D-4CLS, respectively. Such a reduction in the classification accuracy can be attributed to the following two factors. (1) The number of emotion classes considered in the 1D-3CLS and 2D-5CLS is larger than the number of emotion classes considered in the 1D-2CLS and 2D-4CLS, respectively. Hence, it is expected to have a reduction in the classification accuracy as the number of classes is increased. (2) The presence of the neutral class, which corresponds to EEG samples that have arousal and valence values within the interval (3.5–6.5), as a separate class in the 1D-3CLS and 2D-5CLS can be easily confused with the EEG samples that belong to the other classes and have arousal/valence values close to the interval (3.5–6.5) [69]. In fact, Zhuang et al. [62] have shown that the topographic maps of the brain activities associated with the neutral class have lower values compared with the other emotion classes. This in turn increases the difficulty to discriminate between the EEG samples corresponding to the neutral class and the EEG samples of the other classes that have arousal/valence values that are relatively close to the neutral class. The aforementioned factors explain the decrease in the classification accuracies obtained for the emotion labeling schemes that include the neutral class.

In general, Table 6 shows that the results obtained using each combination of emotion labeling scheme and EEG channel configuration are well above the average random classification accuracy, which is defined as the reciprocal of the number of emotion classes. Specifically, the random classification accuracies for 1D-2CLS, 1D-3CLS, 2D-4CLS and 2D-5CLS are 50%, 33.3%, 25% and 20%, respectively.

### 4.2. Feature-Based Evaluation Analysis

**Figure 4:**
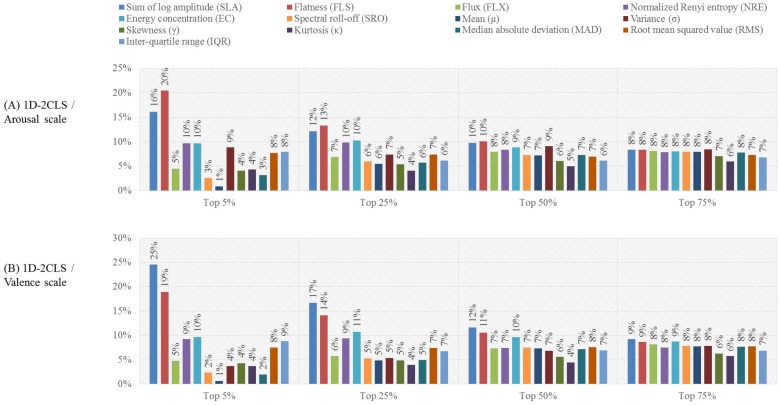
The ratio between the number of times each time-frequency feature is selected to the total number of selected features computed for each of the four feature selection scenarios. (**A**) presents the computed percentages for the 1D-2CLS using the arousal scale, and (**B**) presents the computed percentages for the 1D-2CLS using the valence scale.

**Figure 5:**
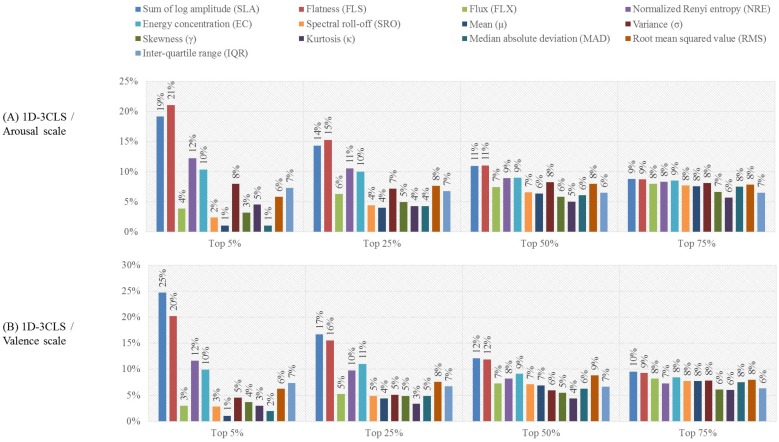
The ratio between the number of times each time-frequency feature is selected to the total number of selected features computed for each of the four feature selection scenarios. (**A**) presents the computed percentages for the 1D-3CLS using the arousal scale, and (**B**) shows the computed percentages for the 1D-3CLS using the valence scale.

**Figure 6:**
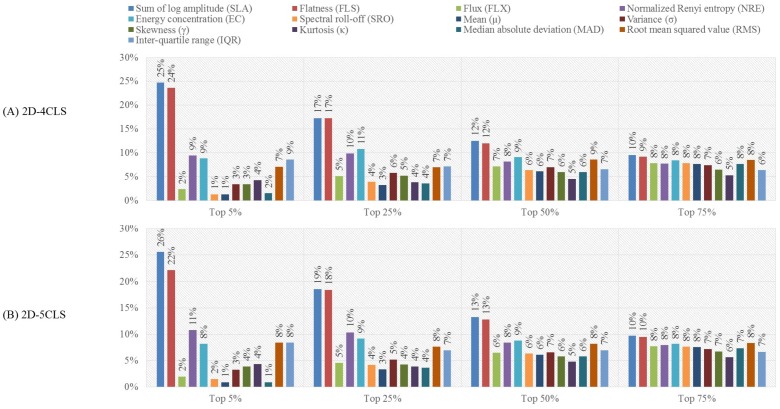
The ratio between the number of times each time-frequency feature is selected to the total number of selected features computed for each of the four feature selection scenarios. (**A**) presents the computed percentages for the 2D-4CLS, and (**B**) shows the computed percentages for the 2D-5CLS.

One can observe that the results presented in Table 7 that were obtained for each emotion labeling scheme using the top 25% features outperform the results of $C_{4}$ presented in Table 6, which were obtained using all features. This implies that a subset of the features extracted from the EEG channels of $C_{4}$ provides salient information that can enhance the classification accuracy. To further investigate the importance of each of the thirteen time-frequency features, for each subject, we computed the ratio between the number of times each time-frequency feature is selected to the total number of selected features in each of the four feature selection scenarios, namely the top 5%, top 25%, top 50% and top 75% scenarios. Then, for each feature selection scenario, we compute the average ratio of each of the thirteen time-frequency features over all 32 subjects. The computed ratios of the thirteen time-frequency features for the 1D-2CLS, 1D-3CLS, 2D-4CLS and 2D-5CLS are shown in Figure 4, Figure 5 and Figure 6, respectively.

The results presented in Figure 4, Figure 5 and Figure 6 indicate that none of the thirteen time-frequency features was completely excluded from the selected subset of features associated with each of the four feature selection scenarios. This can be observed by noticing that the ratio of each of the thirteen time-frequency features computed for each feature selection scenario is strictly larger than zero. This implies that, for each emotion labeling scheme and feature selection scenario, each of the thirteen time-frequency features computed for the EEG channels in $C_{4}$ was included in the selected features for at least one EEG channel. These results suggest that different time-frequency features, which are extracted from different EEG channels, can capture various emotion-related information.

Moreover, for the top 25% feature selection scenario, the ratios computed for each labeling scheme, which are presented in Figure 4, Figure 5 and Figure 6, show that the SLA, FLS, NRE and EC features have the highest selection ratios compared to the other time-frequency features. This can be attributed to the essence of information captured by these features. In particular, the SLA feature is a spectral moment-related feature of the bispectrum of the EEG signals, which captures the nonlinearity of the energy distribution in the constructed CWD-based TFPs of the EEG signals [18,70]. In addition, the FLS feature quantifies the uniformity of the energy distribution of the EEG signals in the constructed CWD-based TFPs [18,71]. Furthermore, the NRE and EC features quantify the regularity and spread, respectively, of the energy distribution in the constructed CWD-based TFPs [18,36,72,73,74].

Its worth noting that the aforementioned four features have performed well compared with the other time-frequency features when used to analyze EEG signals for decoding different motor imagery tasks within the same hand, as indicated in our previous study [18]. This implies that these features are capable of capturing the nonstationary and nonlinear characteristics of the EEG signals, which suggests the feasibility of using the time-frequency features to develop classifiers that target various EEG-based classification problems.

### 4.3. Neutral Class Exclusion Analysis

The average acc and $F_{1}$ values reported in Table 9 and Table 10, which were obtained after excluding the neutral class, are higher than the average acc and $F_{1}$ values reported in Table 6 and Table 7, which were achieved without excluding the neutral class. This finding indicates that the neutral class, which represents the no-emotion class, introduces a large confusion that can significantly reduce the ability to achieve accurate discrimination between the different emotion classes. This can be attributed to the fact that a slight increase or decrease in the emotion rating values provided by the participant can change the class of the emotional state from LA/LV to HA/HV and vice versa, respectively.

**Figure 7:**
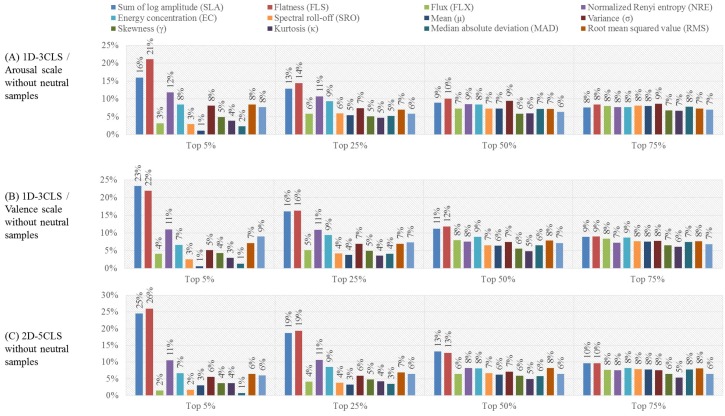
The ratio between the number of times each time-frequency feature is selected to the total number of selected features for each of the four feature selection scenarios after excluding the feature vectors associated with the neutral class. (**A**) presents the computed percentages for the 1D-3CLS using the arousal scale; (**B**) presents the computed percentages for the 1D-3CLS using the valence scale; and (**C**) presents the computed percentages for the 2D-5CLS using the valence scale.

In order to investigate the importance of each of the thirteen time-frequency features, we have computed the ratio between the number of times each time-frequency feature is selected to the total number of selected features for each of the four feature selection scenarios, namely the top 5%, top 25%, top 50% and top 75% scenarios. Figure 7 shows the ratios of the thirteen time-frequency features computed for the 1D-3CLS and 2D-5CLS after excluding the feature vectors that correspond to the neutral class. The results reported in Figure 7 represent the average ration of each of the thirteen time-frequency feature computed over all EEG channels of $C_{4}$ and all 32 participants.

The results presented in Figure 7 indicate that, for the top 25% feature selection scenario, the SLA, FLS, NRE and EC features have the highest selection percentages. These results agree with the evaluations reported in Section 3.2, which implies the importance of the aforementioned time-frequency features in decoding different emotional states. In fact, the results of the feature-based evaluation analysis presented in this subsection, as well as the results presented in Section 3.2 indicate that utilizing all the time-frequency features that are extracted from the 22 EEG channels in $C_{4}$ can degrade the classification performance. This can be attributed to the fact that several features that are extracted from the various EEG channels include redundant and unrelated information. This redundant and unrelated information can produce confusion in the generated feature vectors for different emotion classes, which can reduce the classification performance.

### 4.4. Comparison with Other Studies

Different EEG-based emotion recognition studies have been reported in the literature, where these studies involve various numbers of emotion classes, experimental paradigms and conditions and evaluation procedures and metrics. These variations in the previous studies complicate the task of comparing the performance of our proposed approach with the performance of previous EEG-based emotion recognition approaches. Nonetheless, in this section, we attempt to provide a comparison between the performance of our proposed approach and other existing approaches that utilize the DEAP dataset based on the classification accuracy values reported in previous studies. Moreover, we provide a brief description for some of the existing state-of-the-art EEG-based emotion recognition approaches that utilize the DEAP dataset.

Over the past decade, various approaches have been proposed for emotion recognition based on EEG signals analysis. These approaches can be generally grouped into three categories depending on the number of emotion classes considered in the classification process. In particular, the first category, which represents the vast majority of the existing approaches, utilizes the 1D-2CLS to define two emotion classes for each of the arousal and valence scales, as depicted in Section 2.2. In this vein, Koelstra et al. [24] developed the DEAP dataset, described in Section 2.1, to support emotion analyses using physiological signals. In their study, two emotion classes were defined for each of the arousal and valence scales, namely the HA/LA and HV/LV classes, respectively. In order to discriminate between the emotion classes associated with the arousal and valence scales, two Gaussian naive Bayes classifiers were constructed for each participant to classify the EEG signals into HA/LA and HV/LV, respectively. The classifiers were trained using power spectral features that were extracted from the 32 EEG channels provided as part of the DEAP dataset. The average classification accuracies computed over all the participants in the DEAP dataset were 62% and 57.6% for the arousal and valence scales, respectively. In addition, Rozgic et al. [27] utilized the DEAP dataset to develop an approach for classifying EEG signals into HA/LA and HV/LV classes. In particular, EEG signals were divided into overlapped segments. Then, each EEG segment was analyzed to extract features that are computed using the power spectral density of the different frequency bands comprised within the EEG signals and the difference in the spectral power between 14 symmetrical EEG channels. These features were used to construct three types of classifiers, including the nearest neighbor (NN) voting, naive Bayes NN (NB-NN) and SVM classifiers. The experimental results reported in [27] indicate that the best performance was achieved using the SVM classifier with an average classification accuracies of 69.1% and 76.9% for the arousal and valence scales, respectively. Liu et al. [26] presented an approach for emotion classification in which the EEG signals were represented using deep belief networks (DBNs). The experimental results reported in [26], which were obtained using the DEAP dataset, showed that the average classification accuracies attained in discriminating between the HA/LA and HV/LV classes were 80.5% and 85.2%, respectively. In another study, Li et al. [52] proposed an approach for emotion classification by using weighted fusion SVM classifiers. Specifically, the DEAP dataset was employed to perform the analysis, such that eight EEG channels were utilized to extract time-domain, frequency-domain and nonlinear dynamic domain features. The extracted features were used to train and evaluate the performance of the weighted fusion SVM classifiers. The average classification accuracies achieved in discriminating between the HA/LA and HV/LV classes were 83.7% and 80.7%, respectively. Moreover, Zhuang et al. [25] proposed an approach for emotion recognition based on the empirical mode decomposition (EMD) method. In particular, eight EEG channels out of the 32 EEG channels provided in the DEAP dataset were decomposed into intrinsic mode functions (IMFs) using the EMD method. Then, a set of features was extracted from the computed IMFs, including the first difference of time series, the first difference of phase and the normalized energy features. The extracted features were used to train a set of SVM classifiers to discriminate between the HA/LA and HV/LV classes. The average classification accuracies computed over all the participants in the DEAP dataset were 71.9% and 69.1% for the arousal and valence scales, respectively. Yin et al. [17] proposed an approach for emotion recognition using a multiple-fusion layer-based ensemble classifier of stacked autoencoder (MESAE). Specifically, a set of 425 features was extracted from the philological signals provided in the DEAP dataset. These features were used to construct a set of MESAE classifiers that classify the EEG signals into HA/LA and HV/LV classes. The achieved classification accuracies for the arousal and valence scales were 77.19% and 76.17%, respectively.

In comparison with the results reported in [17,24,25,26,27,52], Table 7 shows that the average $acc/F_{1}$ values of our proposed approach in discriminating between the HA/LA and HV/LV classes were 86.6%/83.8% and 85.8%/82.4%, respectively, using the top 25% feature selection scenario. In fact, these results indicate that the results achieved by our proposed approach outperformed the results reported in [17,24,25,26,27,52].

The second category of previous studies includes emotion classification approaches that utilize the 1D-3CLS to define three emotion classes for each of the arousal and valence scales, namely the HA/neutral/LA and HV/neutral/LV classes. In this category, Chung and Yoon [28] employed the DEAP dataset to construct a set of subject-dependent Bayes classifiers that discriminate between emotion classes defined using the 1D-2CLS and 1D-3CLS. In particular, the Bayes classifiers were trained using spectral power features that were computed for 93 EEG channels, including the 32 EEG channels provided in the DEAP dataset and 61-virtual channels constructed using bipolar montage. The dimensionality of each feature vector was equal to 392 features. The average classification accuracies obtained for the 1D-2CLS based on the arousal and valence scales were 66.4% and 66.6%, and the accuracies achieved for the 1D-3CLS based on the arousal and valence scales were 51.0% and 53.4%, respectively. Similarly, Tripathi et al. [29] employed neural networks to classify the EEG signals provided in the DEAP dataset into two classes using the 1D-2CLS and three classes using the 1D-3CLS. Specifically, statistical time-domain features were extracted from the 32 EEG channels (the dimensionality of each feature vector was 101 features × 32 EEG channels = 3232 features) and used to construct two types of neural networks, namely the simple deep neural network (DNN) and convolutional neural network (CNN). The experimental results reported in [29] showed that the best performance was achieved using the CNN. In particular, for the 1D-2CLS, the average classification accuracies computed over all participants in the DEAP dataset were 73.3% and 81.4% for the arousal and valence scales, respectively. For the 1D-3CLS, the average classification accuracies computed over all participants in the DEAP dataset were 57.5% and 66.7% for the arousal and valence scales, respectively. Atkinson and Campos [31] developed an approach that combines the mRMR feature selection algorithm and SVM classifier for emotion recognition. In particular, a set of features, including statistical features, band power for different frequency bands, Hjorth parameters and fractal dimension, was extracted from 14 EEG channel in the DEAP dataset. The mRMR algorithm was employed to select a subset of the extracted features that achieved the maximum correlation with a specific class and the minimum correlation between the selected features. The dimensionality of each feature vector after applying the mRMR feature selection algorithm was 173 features. The selected subset of features was used to train a set of SVM classifiers to classify the EEG signals into HA/LA and HV/LV classes defined based on the 1D-2CLS or HA/neutral/LA and HV/neutral/LV classes defined based on the 1D-3CLS. For the 1D-2CLS, the average classification accuracies computed for the arousal and valence scales were 73.0% and 73.1%, respectively. Moreover, the average classification accuracies computed for the arousal and valence scales based on the 1D-3CLS were 60.7% and 62.3%, respectively. In another study, Jirayucharoensak et al. [30] proposed an approach for emotion recognition that utilizes a deep learning network (DLN) to study the correlation between the EEG signals. The DLN was trained using spectral-based feature vectors, where the dimensionality of each feature vector was 230 features. The proposed approach was evaluated using the DEAP dataset and achieved average classification accuracies of 49.5% and 46.0% in discriminating between the HA/neutral/LA and HV/neutral/LV classes, respectively. Menezes et al. [4] investigated the use of different combinations of features, including statistical, power spectral density (PSD) and higher order crossings (HOC) features per EEG channel, as well as different classifiers, including the SVM and random forest classifiers, to analyze EEG signals for emotion recognition. The results indicated that the best classification accuracies achieved for the arousal and valence scales based on the 1D-2CLS were 74.0% and 88.4%, respectively. In fact, these accuracies were obtained by combining the statistical features and the SVM classifier. Moreover, for the 1D-3CLS, the best classification accuracies were 63.1% and 58.8% for the arousal and valence scales, respectively, which were obtained by combining the statistical features (the dimensionality of each feature vector was 28 features × 4 EEG channels = 112 features) and the random forest classifier. In comparison with the results presented in the studies [4,28,29,30,31], Table 7 shows that the average $acc/F_{1}$ values achieved by our proposed approach in discriminating between the HA/neutral/LA and HV/neutral/LV classes were 78.8%/65.8% and 77.8%/70.6%, respectively, using the top 25% feature selection scenario. In fact, these results indicate that the performance of our proposed approach outperformed the results reported in [4,28,29,30,31].

The last category of previous studies includes emotion recognition approaches that utilize the 2D-4CLS to define four emotion classes, namely the HAHV, LAHV, LALV and HALV emotion classes. One example of this category is the study by Zheng et al. [32], in which intensive performance evaluation analyses were conducted using different methods for emotion recognition using the EEG signals of the DEAP dataset that integrated various feature extraction, feature selection and pattern classification approaches. The best average classification accuracy achieved in discriminating between the HAHV, LAHV, LALV and HALV emotion classes was 69.6%, which was obtained using the differential entropy features (the dimensionality of each feature vector was 160 features) and the graph regularized extreme learning machine (GELM) classifier. In another study, Zubair and Yoon [33] proposed an EEG-based emotion recognition approach using the discrete wavelet transform (DWT). In particular, a set of features was extracted from the DWT coefficients computed for the EEG signals in the DEAP dataset. Then, the mRMR algorithm was employed to select the most significant subset of features from the extracted DWT-based features. The dimensionality of each feature vector after applying the mRMR feature selection algorithm was 288 features. Using the selected subset of features, a multi-class SVM classifier was constructed for each participant to discriminate between the HAHV, LAHV, LALV and HALV emotion classes. The overall average classification accuracy was 49.7%. Compared with the results presented in the studies [32,33], Table 7 shows that our proposed approach achieved average $acc/F_{1}$ values of 75.1%/68.8% in discriminating between the HAHV, LAHV, LALV and HALV emotion classes using the top 25% feature selection scenario. These results indicate that the performance of our proposed approach outperformed the results reported in [32,33].

To the best of our knowledge, a limited number of studies have investigated the effect of excluding the neutral class on the accuracy of emotion classification. For example, Menezes et al. [4] studied the effect of the neutral class by excluding the neutral samples defined based on the 1D-3CLS. The reported average accuracy in discriminating between the HA/LA and HV/LV classes were 74% and 88.4%, respectively. Compared with the results presented in [4], which were obtained after excluding the neutral samples in the 1D-3CLS, our approach achieved average acc values of 89.8% and 88.9% in discriminating between the HA/LA and HV/LV classes, respectively. These results indicate that our approach is an improvement over the work presented in [4]. In addition to the conventional evaluations of our proposed approach that we have performed based on the 1D-2CLS, 1D-3CLS and 2D-4CLS, the present study took a further step by evaluating the performance of our proposed approach in classifying the five emotion classes defined using the 2D-5CLS. To the best of our knowledge, the 2D-5CLS has not been included in the performance evaluations reported in the previous literature. Table 7 shows that, based on the 2D-5CLS labeling scheme, our proposed approach achieved average $acc/F_{1}$ values of 73.8%/61.9% in discriminating between the HAHV, LAHV, LALV, HALV and neutral emotion classes using the top 25% feature selection scenario. In addition, the effect of excluding the neutral samples in the 2D-5CLS was also investigated. The results presented in Table 10 indicate that our proposed QTFD-based approach achieved average $acc/F_{1}$ values of 79.3%/66.7% in discriminating between the HAHV, LAHV, LALV and HALV emotion classes, which were obtained after excluding the neutral samples, by employing the top 25% feature selection scenario. In fact, the results reported in the current study suggest that the proposed approach provides a promising direction for decoding different emotional states based on analyzing EEG signals using QTFDs. Table 11 provides a summary of the classification accuracies obtained for the aforementioned emotion recognition approaches along with the results obtained using our proposed QTFD-based approach.

### 4.5. Limitations and Future Work

Despite the promising results obtained using our proposed QTFD-based approach in recognizing various emotion classes, there are several future research directions that we are planning to investigate with the goal of improving the robustness, accuracy and applicability of our proposed emotion recognition approach. In particular, we are planning to investigate the following five future research directions:

- Firstly, in this study, we have utilized SVM classifiers to decode various emotion classes. In fact, the selection of the SVM classifier was based on the fact that SVM classifiers have been employed in the vast majority of the existing EEG-based emotion recognition studies, which in turn simplifies the comparison between the performance of our proposed approach and previous approaches. In addition, the SVM classifier has been reported in many previous studies to achieve good classification performance compared with other classifiers. Nonetheless, motivated by the recent promising results attained in analyzing physiological signals using deep learning approaches [75], we are planning to investigate the use of deep learning approaches, such as convolutional neural network (CNN), to extract time-frequency features from the constructed QTFD-based representation. Moreover, we intend to utilize the learned CNN-based time-frequency features with other types of classifiers, such as long short-term memory (LSTM) networks, which may provide a better description of how emotional states evolve over time. In addition, the promising results reported in [12] that were obtained by analyzing multi-modality emotion-related acquisition signals, such as visual, speech and text signals, using a deep learning technique, such as CNN, suggest that utilizing different emotion-related input modalities might improve the emotion classification performance. Therefore, in the future, we intend to employ deep learning techniques to enhance the performance of our approach by analyzing different emotion-related signals acquired using different modalities, including EEG signals, speech and visual cues.
- Secondly, the nonstationary nature of the EEG signals and the inter- and inter-personal variations in emotion responses impose the need to construct a large-scale and well-balanced dataset to avoid classification bias and overfitting problems. Moreover, the recorded physiological responses to stimuli in real-world applications may differ from the responses recorded in a well-controlled environment. This implies that the results presented in this study may overestimate the performance in real-world applications. Therefore, in the near future, we plan to acquire a large-scale EEG dataset under realistic recording conditions to evaluate the effectiveness of our proposed QTFD-based emotion recognition approach in real-world applications.
- Thirdly, our proposed approach, including the computation of the QTFD and the extraction of the thirteen time-frequency features, was implemented using MATLAB (The MathWorks Inc., Natick, MA, USA). The QTFD and the feature extraction routines were executed on a computer workstation with a 3.5-GHz Intel Xeon Processor (Intel Corporation, Santa Clara, CA, USA) and 8 GB memory. The average ± standard deviation time required to compute the QTFD for an EEG segment of length 512 samples is 9.1 ms ± 3.2 ms, and the average ± standard deviation time required to extract the thirteen time-frequency features from the computed QTFD is 34.2 ms ± 4.52 ms. Therefore, the average time required to compute the QTFD for the 22 EEG channels in $C_{4}$ is approximately 200.2 ms, while the average time required to compute the thirteen time-frequency features for the 22 EEG channels in $C_{4}$ is approximately 752.4 ms. Thus, the total time required to compute the QTFD and extract the thirteen time-frequency features for the 22 EEG channels is approximately 952.6 ms, which is less than the duration of the utilized sliding window (i.e., 4 s). This implies that our proposed approach can be used in real-world applications. Nonetheless, we believe that there is still a room to improve the run-time of the proposed approach using parallel computing technology, which allows the utilization of our approach in various clinical applications.
- Fourthly, we are also planning to customize our proposed approach to target specific clinical applications, such as pain detection. In particular, rather than classifying the emotion classes into high versus low arousal/valence levels, which is the main goal of the current study, we plan in the near future to extend our work by utilizing the extracted time-frequency features to estimate the values of the arousal and valence scales associated with various emotional states. Such an extension can be of great benefit for estimating the level of pain a patient is feeling, especially for patients who are unable to verbally communicate their feelings.
- Finally, we plan to extend the analyses conducted in the current study from subject-dependent analyses to subject-independent analyses. Such an extension can provide insight regarding the ability of the proposed QTFD-based approach to recognize emotion classes for new subjects that were not part of the training set, which can be of great benefit for several real-world applications.

## 5. Conclusions

Emotion recognition is an essential task to improve the performance of many human-machine interaction procedures. This study introduces a new EEG-based emotion recognition approach that employs a QTFD, namely the CWD, to construct a TFR of the EEG signals. In order to reduce the dimensionality of the constructed CWD-based TFR, a set of time-frequency features is extracted from the CWD representation of each EEG segment. These features are used to classify different emotion classes, which are defined based on four emotion labeling schemes, using subject-dependent SVM classifiers. A publicly available EEG dataset has been employed to validate the performance of the proposed approach using three different performance evaluation analyses. Moreover, the performance of the proposed approach was compared with the performance reported in several state-of-the-art EEG-based emotion recognition approaches. The results reported in the current study demonstrate the feasibility of our proposed approach to enable accurate emotion recognition based on EEG signal analysis.

## Figures and Tables

**Table 1:** The number of trials and feature vectors per emotion class for each labeling scheme. The table includes the total number of trials for the 32 subjects, the total number of feature vectors (number of trials × number of windows per trial) for the 32 subjects and the mean number of feature vectors (number of trials × number of windows per trial/32 subjects) for each individual subject.

Emotion Labeling Scheme	Emotion Description Scale	Emotion Class	Number of Trials	Number of Feature Vectors for the 32 Subjects	The Mean Number of Feature Vectors for Each Individual Subject
1D-2CLS	Arousal	Low arousal (LA)	543	15,747	492
		High arousal (HA)	737	21,373	668
	Valence	Low valence (LV)	572	16,588	518
		High valence (HV)	708	20,532	518
1D-3CLS	Arousal	Low arousal (LA)	304	8816	276
		Neutral	607	17,603	334
		High arousal (HA)	369	10,701	550
	Valence	Low valence (LV)	297	8613	269
		Neutral	537	15,573	404
		High valence (HV)	446	12,934	487
2D-4CLS	2D arousal-valence plane	HAHV	439	12,731	398
		LAHV	269	7801	244
		LALV	274	7946	248
		HALV	298	8642	270
2D-5CLS	2D arousal-valence plane	HAHV	368	10,672	334
		LAHV	198	5742	179
		LALV	208	6032	189
		HALV	220	6380	199
		Neutral	286	8294	259

**Table 2:** The extracted time-frequency features obtained by extending eight time-domain features to the joint time-frequency-domain. SLA, sum of the logarithmic amplitudes.

Description of the Time-Frequency Features	Mathematical Formulation of the Extracted Time-Frequency Features
The mean of the CWD (μ)	(5) $\mu =\frac{1}{M\times N}\sum_{t=1}^{M}\sum_{f=1}^{N}\varrho_{a}\left(t,f\right)$
The variance of the CWD (σ)	(6) $\sigma =\frac{1}{M\times N}\sum_{t=1}^{M}\sum_{f=1}^{N}{\left(\varrho_{a}\left(t,f\right)-\mu \right)}^{2}$
The skewness of the CWD (γ)	(7) $\gamma =\frac{1}{M\times N\times {\left(\sigma \right)}^{3/2}}\sum_{t=1}^{M}\sum_{f=1}^{N}{\left(\varrho_{a}\left(t,f\right)-\mu \right)}^{3}$
The kurtosis of the CWD (κ)	(8) $\kappa =\frac{1}{M\times N\times {\left(\sigma \right)}^{2}}\sum_{t=1}^{M}\sum_{f=1}^{N}{\left(\varrho_{a}\left(t,f\right)-\mu \right)}^{4}$
Sum of the logarithmic amplitudes of the CWD (SLA)	(9) $SLA=\sum_{t=1}^{M}\sum_{f=1}^{N}\log(|\varrho_{a}\left(t,f\right)\left|\right)$
Median absolute deviation of the CWD (MAD)	(10) $MAD=\frac{1}{M\times N}\sum_{t=1}^{M}\sum_{f=1}^{N}\left|\varrho_{a}\left(t,f\right)-\mu \right|$
Root mean square value of the CWD (RMS)	(11) $RMS=\sqrt{\left(\sum_{t=1}^{M}\sum_{f=1}^{N}\varrho_{a}\left(t,f\right)\right)/\left(M\times N\right)}$
Inter-quartile range of the CWD (IQR)	(12) $IQR=\frac{1}{N}\sum_{f=1}^{N}\left(\varrho_{a}\left(\frac{3(M+1)}{4},f\right)-\varrho_{a}\left(\frac{\left(M+1\right)}{4},f\right)\right)$

**Table 3:** The extracted time-frequency features obtained by extending five frequency-domain features to the joint time-frequency-domain.

Description of the Time-Frequency Features	Mathematical Formulation of the Extracted Time-Frequency Features
The flatness of the CWD (FLS)	(13) $FLS=M\times N\times \frac{\prod_{t=1}^{M}\prod_{f=1}^{N}\left(\right|\varrho_{a}\left(t,f\right){\left|\right)}^{1/M\times N}}{\sum_{t=1}^{M}\sum_{f=1}^{N}\left(\right|\varrho_{a}\left(t,f\right)\left|\right)}$
The flux of the CWD (FLX)	(14) $FLX=\sum_{t=1}^{M-l}\sum_{f=1}^{N-k}\left(\varrho_{a}\left(t+l,f+k\right)-\varrho_{a}\left(t,f\right)\right),l=k=1$
The spectral roll-off of the CWD (SRO)	(15) $SRO=\lambda \sum_{t=1}^{M}\sum_{f=1}^{N}\left(\varrho_{a}\left(t,f\right)\right),\lambda =0.85$
The normalized Renyi entropy of the CWD (NRE)	(16) $NRE=\frac{1}{1-\nu }\log_{2}\sum_{t=1}^{M}\sum_{f=1}^{N}{\left(\frac{\varrho_{a}\left(t,f\right)}{M\times N\times \mu }\right)}^{\nu },\nu =3$
The energy concentration of the CWD (EC)	(17) $EC={\left(\sum_{t=1}^{M}\sum_{f=1}^{N}\sqrt{|\varrho_{a}\left(t,f\right)|}\right)}^{2}$

**Table 4:** The brain regions covered by the selected 11 pairs of EEG channels.

Brain Region	Selected Pairs of EEG Channels
Parietal region	P3-P4, P7-P8 and CP5-CP6
Frontal region	F3-F4, F7-F8, FC1-FC2, FC5-FC6, AF3-AF4 and FP1-FP2
Temporal region	T7-T8
Occipital region	O1-O2

**Table 5:** The four configurations of EEG channels considered in this study.

Configuration	Description	EEG Channels
Configuration 1 ($C_{1}$)	This configuration includes 11 independent pairs of symmetric EEG channels.	P3-P4, P7-P8, CP5-CP6, F3-F4, F7-F8, FC1-FC2, FC5-FC6, AF3-AF4, FP1-FP2, T7-T8, and O1-O2
Configuration 2 ($C_{2}$)	This configuration includes 12 EEG channels located in the frontal and temporal areas of the brain.	FP1, FP2, F3, F4, F7, F8, FC1, FC2, T7, T8, FC5, and FC6
Configuration 3 ($C_{3}$)	This configuration includes eight EEG channels located in the parietal and occipital areas of the brain.	P3, P4, CP5, CP6, P7, P8, O1, and O2
Configuration 4 ($C_{4}$)	This configuration includes all the selected EEG channels.	P3, P4, P7, P8, CP5, CP6, F3, F4, F7, F8, FC1, FC2, FC5, FC6, AF3, AF4, FP1, FP2, T7, T8, O1, and O2

**Table 6:** Results of the channel-based evaluation analysis. Bold font is used to indicate the highest acc and $F_{1}$ values obtained for each combination of emotion labeling scheme and EEG channel configuration.

Configuration		1D-2CLS				1D-3CLS				2D-4CLS		2D-5CLS	
		Arousal		Valence		Arousal		Valence					
		acc	$F_{1}$	acc	$F_{1}$	acc	$F_{1}$	acc	$F_{1}$	acc	$F_{1}$	acc	$F_{1}$
C1	AF3-AF4	72.5	62.3	73.9	69.7	65.2	41.2	64.0	49.2	58.4	45.9	56.4	42.0
	CP5-CP6	74.4	63.1	71.7	66.6	65.4	41.8	64.0	49.8	58.1	44.5	55.5	40.5
	F3-F4	73.7	63.0	71.6	67.0	65.1	39.6	63.0	48.3	57.1	45.4	55.0	41.0
	F7-F8	74.3	64.1	72.8	68.4	65.8	41.2	64.3	48.9	58.9	46.8	56.9	42.8
	FC1-FC2	73.0	61.7	72.1	66.4	64.6	40.1	62.4	46.9	57.1	44.8	54.9	40.4
	FC5-FC6	74.7	65.1	73.5	68.7	66.2	42.0	64.7	51.3	59.5	46.4	57.2	43.1
	FP1-FP2	74.3	64.1	71.6	66.7	65.9	42.0	63.1	49.6	58.0	48.1	55.5	41.8
	O1-O2	75.9	66.7	72.8	67.4	65.9	42.1	63.2	49.8	58.7	45.9	56.1	40.1
	P3-P4	74.7	64.6	72.4	67.3	65.6	41.3	64.1	48.9	58.7	45.5	55.8	41.4
	P7-P8	75.2	64.7	72.0	66.6	66.0	43.0	64.4	50.6	58.6	46.9	57.1	41.3
	T7-T8	74.5	64.6	73.5	69.0	67.0	44.9	65.6	51.6	60.5	49.4	57.9	45.3
C2		81.0	75.1	79.6	77.3	74.7	60.4	73.4	65.3	70.6	62.5	68.8	56.6
C3		80.1	74.4	77.1	73.9	72.1	55.4	70.2	61.2	66.1	56.9	64.9	51.4
C4		**83.1**	**78.5**	**80.7**	**78.5**	**76.0**	**62.2**	**75.6**	**68.1**	**72.5**	**65.0**	**71.1**	**59.7**

**Table 7:** Results of the feature-based evaluation analysis using the EEG channels of $C_{4}$. Bold font is used to indicate the highest acc and $F_{1}$ values obtained for each EEG channel configuration.

Labeling Scheme		Top 5%		Top 25%		Top 50%		Top 75%	
		acc	$F_{1}$	acc	$F_{1}$	acc	$F_{1}$	acc	$F_{1}$
1D-2CLS	Arousal	80.5	73.6	**86.6**	**83.8**	83.7	79.5	83.5	78.7
	Valence	78.7	76.1	**85.8**	**82.4**	82.1	80.6	81.0	79.3
1D-3CLS	Arousal	75.4	58.9	**78.8**	**65.8**	78.4	65.5	77.9	65.0
	Valence	73.7	66.1	**77.8**	**70.6**	76.5	69.4	75.9	68.9
2D-4CLS		69.5	60.9	**75.1**	**68.8**	73.9	66.7	73.0	65.5
2D-5CLS		69.9	57.8	**73.8**	**61.9**	73.1	61.0	72.0	60.0

**Table 8:** The accuracy and standard deviation values computed for each subject using the top 25% of the features extracted form the EEG channels of $C_{4}$. STD represents the standard deviation.

Subject	1D-2CLS				1D-3CLS				2D-4CLS		2D-5CLS	
	Arousal		Valence		Arousal		Valence					
	Acc	STD	Acc	STD	Acc	STD	Acc	STD	Acc	STD	Acc	STD
S1	84.9	2.4	83.7	2.6	78.4	2.5	77.3	2.5	76.2	3.1	75.7	4.7
S2	82.3	2.2	81.7	2.9	77.6	1.0	75.8	4.4	73.1	3.0	72.5	2.4
S3	87.9	1.5	86.3	3.4	83.4	2.4	81.2	2.1	78.4	3.1	76.4	1.2
S4	85.3	4.2	84.6	1.5	76.0	4.4	77.9	2.0	75.2	3.2	73.8	3.9
S5	90.4	2.2	91.0	3.2	77.6	1.8	78.6	0.4	74.0	3.5	72.9	3.1
S6	82.4	1.7	81.9	1.4	78.0	2.4	77.0	2.1	74.2	4.2	72.8	1.1
S7	87.5	1.4	88.7	2.1	86.6	2.5	86.1	1.2	84.3	1.4	82.3	1.5
S8	88.8	0.8	87.3	2.0	75.0	1.9	73.8	0.9	71.5	4.7	70.7	1.5
S9	85.3	1.0	86.2	2.7	84.0	2.6	83.2	1.6	81.0	2.3	80.9	2.0
S10	88.6	1.6	87.2	2.1	85.6	1.9	83.4	1.5	78.4	3.1	76.6	2.9
S11	86.0	1.8	84.3	2.2	72.3	3.3	71.0	2.3	67.7	2.1	65.7	2.7
S12	84.5	1.8	82.7	2.2	75.4	2.3	73.5	3.0	68.1	2.0	66.8	1.7
S13	92.2	0.9	91.8	2.8	82.4	2.8	81.9	4.3	77.4	2.4	75.8	1.9
S14	86.3	2.7	85.5	1.0	75.5	3.2	74.1	2.5	70.1	2.2	69.7	2.0
S15	84.8	1.7	83.1	1.3	80.9	1.8	81.3	2.0	79.1	2.7	77.9	2.7
S16	92.7	5.0	91.4	2.4	86.6	2.2	84.9	1.8	82.3	3.5	81.7	1.3
S17	86.5	3.1	85.0	3.8	83.5	1.7	82.3	1.8	80.0	1.6	78.0	1.6
S18	84.4	4.3	86.1	2.6	81.6	2.7	83.6	2.8	80.2	1.5	78.4	2.7
S19	82.6	3.2	82.8	2.7	74.6	2.7	74.1	3.7	72.2	2.0	70.9	1.9
S20	85.8	3.2	83.2	3.7	75.9	1.4	73.3	2.8	71.3	1.7	69.1	5.0
S21	86.2	2.7	85.2	4.4	72.4	2.2	71.4	1.5	69.1	2.5	67.9	1.9
S22	85.4	1.2	84.6	2.0	75.1	2.5	73.4	3.5	70.2	6.6	68.9	4.1
S23	91.8	1.5	90.5	2.3	86.2	2.6	87.9	1.0	83.6	3.3	81.6	2.2
S24	87.9	3.5	85.4	3.3	76.4	2.6	75.3	2.7	73.2	1.3	71.8	1.7
S25	84.6	3.1	83.6	2.5	74.3	2.1	72.8	2.7	69.2	3.7	68.3	3.7
S26	87.7	2.4	86.6	2.3	78.8	2.0	73.2	4.1	70.2	2.1	69.2	4.0
S27	83.8	1.3	82.3	1.9	76.6	4.7	72.4	1.9	78.2	3.2	76.3	2.3
S28	92.7	2.9	91.7	3.3	79.9	2.0	79.8	2.1	77.2	2.7	73.9	2.0
S29	84.3	2.7	84.0	2.7	80.1	1.6	78.1	2.8	74.2	1.9	72.8	2.3
S30	86.6	3.2	85.9	2.4	82.7	1.2	81.5	2.7	79.0	2.4	77.6	3.0
S31	84.2	0.8	83.7	3.5	75.1	3.1	73.7	1.1	72.1	2.1	70.9	4.4
S32	88.0	2.2	87.6	1.0	73.3	1.2	75.0	2.4	73.3	4.3	72.0	2.8
Overall average	86.6	2.3	85.8	2.5	78.8	2.4	77.8	2.3	75.1	2.8	73.7	2.6

**Table 9:** The results of the channel-based evaluation analysis computed for the 1D-3CLS and 2D-5CLS after excluding the feature vectors that correspond to the neutral class. Bold font is used to indicate the highest acc and $F_{1}$ values obtained for each combination of emotion labeling scheme and EEG channel configuration.

Configuration		1D-3CLS				2D-5CLS	
		Arousal		Valence			
		acc	$F_{1}$	acc	$F_{1}$	acc	$F_{1}$
C1	AF3-AF4	81.5	70.7	79.5	71.5	64.2	49.2
	CP5-CP6	79.7	67.5	79.4	70.1	63.8	47.3
	F3-F4	79.7	67.6	78.2	68.5	62.3	47.2
	F7-F8	80.6	69.9	79.0	70.9	64.4	49.5
	FC1-FC2	80.7	68.9	78.5	68.6	63.5	47.2
	FC5-FC6	80.4	68.7	79.7	70.7	64.3	50.0
	FP1-FP2	81.4	69.9	79.1	70.7	63.2	48.1
	O1-O2	80.1	68.2	79.2	71.1	63.7	46.6
	P3-P4	81.3	69.4	78.8	70.0	63.9	47.8
	P7-P8	80.4	69.6	79.4	69.1	64.4	49.1
	T7-T8	82.2	71.4	80.1	72.2	65.4	50.1
C2		87.4	78.6	85.6	79.8	74.0	60.3
C3		85.2	76.1	83.5	76.5	70.5	56.8
C4		**88.4**	**80.2**	**87.0**	**81.9**	**77.0**	**63.8**

**Table 10:** The results of the feature-based evaluation analysis computed for the 1D-3CLS and 2D-5CLS after excluding the feature vectors that correspond to the neutral class. Bold font is used to indicate the highest acc and $F_{1}$ values obtained for each EEG channel configuration.

Labeling Scheme		Top 5%		Top 25%		Top 50%		Top 75%	
		acc	$F_{1}$	acc	$F_{1}$	acc	$F_{1}$	acc	$F_{1}$
1D-3CLS	Arousal	84.6	78.4	**89.8**	**81.8**	88.6	80.8	88.5	80.5
	Valence	85.9	80.1	**88.9**	**83.1**	87.7	82.4	87.0	81.9
2D-5CLS		74.2	60.4	**79.3**	**66.7**	78.9	66.3	77.9	65.1

**Table 11:** Comparison of classification accuracies obtained for various previous approaches. EMD, empirical mode decomposition; QTFD, quadratic time-frequency distribution; HOC, higher order crossings.

Method	Features and Classifier	Number of EEG Channels	Labeling Scheme	Accuracy (%)	
				Arousal	Valence
Koelstra et al. [24], 2012	Power spectral features, Gaussian naive Bayes classifier	32	1D-2CLS	62.0	57.6
Chung and Yoon [28], 2012	Power spectral features, Bayes classifier	32	1D-2CLS	66.4	66.6
Rozgic et al. [27], 2013	Power spectral features, SVM	32	1D-2CLS	69.1	76.9
Liu et al. [26], 2016	Deep belief network-based features, SVM	32	1D-2CLS	80.5	85.2
Atkinson and Campos [31], 2016	Statistical, fractal dimension and band power features, SVM	14	1D-2CLS	73.0	73.1
Tripathi et al. [29], 2017	Statistical time-domain features, neural networks	32	1D-2CLS	73.3	81.4
Zhuang et al. [25], 2017	EMD-based features, SVM	8	1D-2CLS	71.9	69.1
Li et al. [52], 2017	Time, frequency and nonlinear dynamic features, SVM	8	1D-2CLS	83.7	80.7
Yin et al. [17], 2017	Statistical and power spectral features, neural networks	32	1D-2CLS	77.1	76.1
**Our approach**	**QTFD-based features, SVM**	**22**	1D-2CLS	**86.6**	**85.8**
Menezes et al. [4], 2017	Statistical, Power spectral and HOC features, SVM	4	1D-3CLS after excluding the neutral samples	74	88.4
**Our approach**	**QTFD-based features, SVM**	**22**	1D-3CLS after excluding the neutral samples	**89.8**	**88.9**
Chung and Yoon [28], 2012	Power spectral, Bayes classifier	32	1D-3CLS	51.0	53.4
Jirayucharoensak et al. [30], 2014	Principle component analysis, deep leaning network	32	1D-3CLS	52.0	53.4
Atkinson and Campos [31], 2016	Statistical, fractal dimension and band power features, SVM	14	1D-3CLS	60.7	62.3
Menezes et al. [4], 2017	Statistical, power spectral and HOC features, SVM	4	1D-3CLS	63.1	58.8
Tripathi et al. [29], 2017	Statistical time-domain features, neural networks	32	1D-3CLS	57.5	66.7
**Our approach**	**QTFD-based features, SVM**	**22**	1D-3CLS	**78.8**	**77.8**
Zheng et al. [32], 2017	STFT-based features, SVM	32	2D-4CLS	69.6	
Zubair and Yoon [33], 2018	Statistical and wavelet-based features, SVM	32	2D-4CLS	49.7	
**Our approach**	**QTFD-based features, SVM**	**22**	2D-4CLS	**75.1**	
**Our approach**	**QTFD-based features, SVM**	**22**	2D-5CLS after excluding the neutral samples	**79.3**	
**Our approach**	**QTFD-based features, SVM**	**22**	2D-5CLS	**73.8**	

## References

[1] Doukas C., Maglogiannis I. (2008). Intelligent pervasive healthcare systems. Advanced Computational Intelligence Paradigms in Healthcare-3.

[2] Petrantonakis P.C., Hadjileontiadis L.J. (2010). Emotion Recognition From EEG Using Higher Order Crossings. IEEE Trans. Inf. Technol. Biomed..

[3] Purnamasari P.D., Ratna A.A.P., Kusumoputro B. (2017). Development of Filtered Bispectrum for EEG Signal Feature Extraction in Automatic Emotion Recognition Using Artificial Neural Networks. Algorithms.

[4] Menezes M.L.R., Samara A., Galway L., Sant’Anna A., Verikas A., Alonso-Fernandez F., Wang H., Bond R. (2017). Towards emotion recognition for virtual environments: an evaluation of eeg features on benchmark dataset. Pers. Ubiquitous Comput..

[5] Chen J., Hu B., Moore P., Zhang X., Ma X. (2015). Electroencephalogram-based emotion assessment system using ontology and data mining techniques. Appl. Soft Comput..

[6] Bourel F., Chibelushi C.C., Low A.A. Robust facial expression recognition using a state-based model of spatially-localised facial dynamics. Proceedings of the Fifth IEEE International Conference on Automatic Face and Gesture Recognition.

[7] Cohen I., Sebe N., Garg A., Chen L.S., Huang T.S. (2003). Facial expression recognition from video sequences: Temporal and static modeling. Comput. Vis. Image Underst..

[8] Alazrai R., Lee C.G. Real-time emotion identification for socially intelligent robots. Proceedings of the IEEE International Conference on Robotics and Automation (ICRA).

[9] Alazrai R., Lee C.G. An narx-based approach for human emotion identification. Proceedings of the IEEE/RSJ International Conference on Intelligent Robots and Systems (IROS).

[10] Schuller B., Reiter S., Muller R., Al-Hames M., Lang M., Rigoll G. Speaker independent speech emotion recognition by ensemble classification. Proceedings of the IEEE International Conference on Multimedia and Expo.

[11] Yu F., Chang E., Xu Y.Q., Shum H.Y. (2001). Emotion detection from speech to enrich multimedia content. Pacific-Rim Conference on Multimedia.

[12] Poria S., Chaturvedi I., Cambria E., Hussain A. Convolutional MKL based multimodal emotion recognition and sentiment analysis. Proceedings of the IEEE 16th International Conference on Data Mining (ICDM).

[13] Picard R.W., Vyzas E., Healey J. (2001). Toward machine emotional intelligence: Analysis of affective physiological state. IEEE Trans. Pattern Anal. Mach. Intell..

[14] Nasoz F., Alvarez K., Lisetti C.L., Finkelstein N. Emotion recognition from physiological signals for user modeling of affect. Proceedings of the UM 2003, 9th International Conference on User Model.

[15] Nie D., Wang X.W., Shi L.C., Lu B.L. EEG-based emotion recognition during watching movies. Proceedings of the 5th International IEEE/EMBS Conference on Neural Engineering (NER).

[16] Martini N., Menicucci D., Sebastiani L., Bedini R., Pingitore A., Vanello N., Milanesi M., Landini L., Gemignani A. (2012). The dynamics of EEG gamma responses to unpleasant visual stimuli: From local activity to functional connectivity. NeuroImage.

[17] Yin Z., Zhao M., Wang Y., Yang J., Zhang J. (2017). Recognition of Emotions Using Multimodal Physiological Signals and an Ensemble Deep Learning Model. Comput. Methods Prog. Biomed..

[18] Alazrai R., Alwanni H., Baslan Y., Alnuman N., Daoud M.I. (2017). EEG-Based Brain-Computer Interface for Decoding Motor Imagery Tasks within the Same Hand Using Choi-Williams Time-Frequency Distribution. Sensors.

[19] Nicolas-Alonso L.F., Gomez-Gil J. (2012). Brain computer interfaces, a review. Sensors.

[20] Castiglioni P. (2005). Choi-Williams Distribution. Encyclopedia of Biostatistics.

[21] Boubchir L., Al-Maadeed S., Bouridane A. On the use of time-frequency features for detecting and classifying epileptic seizure activities in non-stationary EEG signals. Proceedings of the 2014 IEEE International Conference on Acoustics, Speech and Signal Processing (ICASSP).

[22] Tzallas A.T., Tsipouras M.G., Fotiadis D.I. (2009). Epileptic seizure detection in EEGs using time-frequency analysis. IEEE Trans. Inf. Technol. Biomed..

[23] Russell J.A. (1980). A circumplex model of affect. J. Personal. Soc. Psychol..

[24] Koelstra S., Muhl C., Soleymani M., Lee J.S., Yazdani A., Ebrahimi T., Pun T., Nijholt A., Patras I. (2012). DEAP: A Database for Emotion Analysis; Using Physiological Signals. IEEE Trans. Affect. Comput..

[25] Zhuang N., Zeng Y., Tong L., Zhang C., Zhang H., Yan B. (2017). Emotion Recognition from EEG Signals Using Multidimensional Information in EMD Domain. BioMed Res. Int..

[26] Liu W., Zheng W.L., Lu B.L. (2016). Multimodal emotion recognition using multimodal deep learning. arXiv.

[27] Rozgic V., Vitaladevuni S.N., Prasad R. Robust EEG emotion classification using segment level decision fusion. Proceedings of the 2013 IEEE International Conference on Acoustics, Speech and Signal Processing.

[28] Chung S.Y., Yoon H.J. Affective classification using Bayesian classifier and supervised learning. Proceedings of the 2012 12th International Conference on Control, Automation and Systems.

[29] Tripathi S., Acharya S., Sharma R.D., Mittal S., Bhattacharya S. Using Deep and Convolutional Neural Networks for Accurate Emotion Classification on DEAP Dataset. Proceedings of the Twenty-Ninth AAAI Conference on Innovative Applications.

[30] Jirayucharoensak S., Pan-Ngum S., Israsena P. (2014). EEG-based emotion recognition using deep learning network with principal component based covariate shift adaptation. Sci. World J..

[31] Atkinson J., Campos D. (2016). Improving BCI-based emotion recognition by combining EEG feature selection and kernel classifiers. Expert Syst. Appl..

[32] Zheng W.L., Zhu J.Y., Lu B.L. (2017). Identifying Stable Patterns over Time for Emotion Recognition from EEG. IEEE Trans. Affect. Comput..

[33] Zubair M., Yoon C., Kim K.J., Kim H., Baek N. (2018). EEG Based Classification of Human Emotions Using Discrete Wavelet Transform. IT Convergence and Security 2017.

[34] Niedermeyer E., da Silva F.L. (2005). Electroencephalography: Basic Principles, Clinical Applications, and Related Fields.

[35] Toole J.M.O. (2009). Discrete Quadratic Time-Frequency Distributions: Definition, Computation, and a Newborn Electroencephalogram Application. Ph.D. Thesis.

[36] Boashash B. (2015). Time-Frequency Signal Analysis and Processing: A Comprehensive Reference.

[37] Alazrai R., Aburub S., Fallouh F., Daoud M.I. EEG-based BCI system for classifying motor imagery tasks of the same hand using empirical mode decomposition. Proceedings of the 10th IEEE International Conference on Electrical and Electronics Engineering (ELECO).

[38] Koenig W., Dunn H.K., Lacy L.Y. (1946). The Sound Spectrograph. J. Acoust. Soc. Am..

[39] Mallat S. (2008). A Wavelet Tour of Signal Processing: The Sparse Way.

[40] Boashash B., Ouelha S. (2016). Automatic signal abnormality detection using time-frequency features and machine learning: A newborn EEG seizure case study. Knowl. Based Syst..

[41] Boashash B., Azemi G., O’Toole J.M. (2013). Time-Frequency Processing of Nonstationary Signals: Advanced TFD Design to Aid Diagnosis with Highlights from Medical Applications. IEEE Signal Process. Mag..

[42] Hahn S.L. (1996). Hilbert Transforms in Signal Processing.

[43] Choi H.I., Williams W.J. (1989). Improved time-frequency representation of multicomponent signals using exponential kernels. IEEE Trans. Acoust. Speech Signal Process..

[44] Swami A., Mendel J., Nikias C. (2000). Higher-Order Spectra Analysis (HOSA) Toolbox, Version 2.0.3.

[45] Zheng W.L., Lu B.L. (2015). Investigating Critical Frequency Bands and Channels for EEG-Based Emotion Recognition with Deep Neural Networks. IEEE Trans. Auton. Ment. Dev..

[46] Boashash B., Boubchir L., Azemi G. (2012). A methodology for time-frequency image processing applied to the classification of non-stationary multichannel signals using instantaneous frequency descriptors with application to newborn EEG signals. EURASIP J. Adv. Signal Process..

[47] Qian H., Mao Y., Xiang W., Wang Z. (2010). Recognition of human activities using SVM multi-class classifier. Pattern Recognit. Lett..

[48] Kreßel U.H.G. (1999). Pairwise classification and support vector machines. Advances in Kernel Methods.

[49] Hsu C.W., Lin C.J. (2002). A comparison of methods for multiclass support vector machines. IEEE Trans. Neural Netw..

[50] Chang C.C., Lin C.J. (2011). LIBSVM: A library for support vector machines. ACM Trans. Intell. Syst. Technol..

[51] Zhang J., Zhao S., Huang W., Hu S., Liu D., Xie S., Li Y., Zhao D., El-Alfy E.S.M. (2017). Brain Effective Connectivity Analysis from EEG for Positive and Negative Emotion. Neural Information Processing.

[52] Li X., Yan J.Z., Chen J.H. Channel Division Based Multiple Classifiers Fusion for Emotion Recognition Using EEG Signals. Proceedings of the 2017 International Conference on Information Science and Technology.

[53] Petrantonakis P.C., Hadjileontiadis L.J. (2011). A Novel Emotion Elicitation Index Using Frontal Brain Asymmetry for Enhanced EEG-Based Emotion Recognition. IEEE Trans. Inf. Technol. Biomed..

[54] Coan J.A., Allen J.J., Harmon-Jones E. (2001). Voluntary facial expression and hemispheric asymmetry over the frontal cortex. Psychophysiology.

[55] Khezri M., Firoozabadi M., Sharafat A.R. (2015). Reliable emotion recognition system based on dynamic adaptive fusion of forehead biopotentials and physiological signals. Comput. Methods Programs Biomed..

[56] Niemic C.P., Warren K. (2002). Studies of Emotion.

[57] Lin Y.P., Wang C.H., Jung T.P., Wu T.L., Jeng S.K., Duann J.R., Chen J.H. (2010). EEG-Based Emotion Recognition in Music Listening. IEEE Trans. Biomed. Eng..

[58] Mohammadi Z., Frounchi J., Amiri M. (2017). Wavelet-based emotion recognition system using EEG signal. Neural Comput. Appl..

[59] Ding C., Peng H. (2005). Minimum redundancy feature selection from microarray gene expression data. J. Bioinform. Comput. Biol..

[60] Radovic M., Ghalwash M., Filipovic N., Obradovic Z. (2017). Minimum redundancy maximum relevance feature selection approach for temporal gene expression data. BMC Bioinform..

[61] Jenke R., Peer A., Buss M. (2014). Feature Extraction and Selection for Emotion Recognition from EEG. IEEE Trans. Affect. Comput..

[62] Zhuang N., Zeng Y., Yang K., Zhang C., Tong L., Yan B. (2018). Investigating Patterns for Self-Induced Emotion Recognition from EEG Signals. Sensors.

[63] Zhang J., Chen M., Zhao S., Hu S., Shi Z., Cao Y. (2016). Relieff-based EEG sensor selection methods for emotion recognition. Sensors.

[64] Alazrai R., Momani M., Daoud M.I. (2017). Fall Detection for Elderly from Partially Observed Depth-Map Video Sequences Based on View-Invariant Human Activity Representation. Appl. Sci..

[65] Alazrai R., Momani M., Khudair H.A., Daoud M.I. (2017). EEG-based tonic cold pain recognition system using wavelet transform. Neural Comput. Appl..

[66] Yoon H.J., Chung S.Y. (2013). EEG-based emotion estimation using Bayesian weighted-log-posterior function and perceptron convergence algorithm. Comput. Biol. Med..

[67] Van den Broek S.P., Reinders F., Donderwinkel M., Peters M. (1998). Volume conduction effects in EEG and MEG. Electroencephalogr. Clin. Neurophysiol..

[68] Liao K., Xiao R., Gonzalez J., Ding L. (2014). Decoding individual finger movements from one hand using human EEG signals. PLoS ONE.

[69] Verma G.K., Tiwary U.S. (2016). Affect representation and recognition in 3D continuous valence-arousal-dominance space. Multimed. Tools Appl..

[70] Zhou S.M., Gan J.Q., Sepulveda F. (2008). Classifying mental tasks based on features of higher-order statistics from EEG signals in brain-computer interface. Inf. Sci..

[71] Boashash B., Azemi G., Khan N.A. (2015). Principles of time-frequency feature extraction for change detection in non-stationary signals: Applications to newborn EEG abnormality detection. Pattern Recognit..

[72] Stanković L. (2001). A measure of some time-frequency distributions concentration. Signal Process..

[73] Acharya U.R., Fujita H., Sudarshan V.K., Bhat S., Koh J.E. (2015). Application of entropies for automated diagnosis of epilepsy using EEG signals: A review. Knowl. Based Syst..

[74] Acharya U.R., Fujita H., Sudarshan V.K., Oh S.L., Adam M., Koh J.E., Tan J.H., Ghista D.N., Martis R.J., Chua C.K. (2016). Automated detection and localization of myocardial infarction using electrocardiogram: A comparative study of different leads. Knowl. Based Syst..

[75] Faust O., Hagiwara Y., Hong T.J., Lih O.S., Acharya U.R. (2018). Deep learning for healthcare applications based on physiological signals: A review. Comput. Methods Programs Biomed..

